# Application of Statistical Learning to Identify Omicron Mutations in SARS-CoV-2 Viral Genome Sequence Data From Populations in Africa and the United States

**DOI:** 10.1001/jamanetworkopen.2022.30293

**Published:** 2022-09-07

**Authors:** Lue Ping Zhao, Terry P. Lybrand, Peter Gilbert, Margaret Madeleine, Thomas H. Payne, Seth Cohen, Daniel E. Geraghty, Keith R. Jerome, Lawrence Corey

**Affiliations:** 1 Public Health Sciences Division, Fred Hutchinson Cancer Research Center, Seattle, Washington; 2 Quintepa Computing LLC, Nashville, Tennessee; 3 Department of Chemistry, Vanderbilt University, Nashville, Tennessee; 4 Vaccine and Infectious Disease Division, Fred Hutchinson Cancer Research Center, Seattle, Washington; 5 Department of Medicine, University of Washington School of Medicine, Seattle; 6 Clinical Research Division, Fred Hutchinson Cancer Research Center, Seattle, Washington

## Abstract

**Importance:**

With timely collection of SARS-CoV-2 viral genome sequences, it is important to apply efficient data analytics to detect emerging variants at the earliest time.

**Objective:**

To evaluate the application of a statistical learning strategy (SLS) to improve early detection of novel SARS-CoV-2 variants using viral sequence data from global surveillance.

**Design, Setting, and Participants:**

This case series applied an SLS to viral genomic sequence data collected from 63 686 individuals in Africa and 531 827 individuals in the United States with SARS-CoV-2. Data were collected from January 1, 2020, to December 28, 2021.

**Main Outcomes and Measures:**

The outcome was an indicator of Omicron variant derived from viral sequences. Centering on a temporally collected outcome, the SLS used the generalized additive model to estimate locally averaged Omicron caseload percentages (OCPs) over time to characterize Omicron expansion and to estimate when OCP exceeded 10%, 25%, 50%, and 75% of the caseload. Additionally, an unsupervised learning technique was applied to visualize Omicron expansions, and temporal and spatial distributions of Omicron cases were investigated.

**Results:**

In total, there were 2698 cases of Omicron in Africa and 12 141 in the United States. The SLS found that Omicron was detectable in South Africa as early as December 31, 2020. With 10% OCP as a threshold, it may have been possible to declare Omicron a variant of concern as early as November 4, 2021, in South Africa. In the United States, the application of SLS suggested that the first case was detectable on November 21, 2021.

**Conclusions and Relevance:**

The application of SLS demonstrates how the Omicron variant may have emerged and expanded in Africa and the United States. Earlier detection could help the global effort in disease prevention and control. To optimize early detection, efficient data analytics, such as SLS, could assist in the rapid identification of new variants as soon as they emerge, with or without lineages designated, using viral sequence data from global surveillance.

## Key Points

**Question:**

Could the SARS-CoV-2 Omicron variant have been detected earlier with existing surveillance data and a state-of-the-art statistical learning strategy?

**Findings:**

In this case series of 2698 Omicron cases in Africa and 12 141 Omicron cases in the United States, a statistical learning strategy found that Omicron was dynamically expanding in Africa and the United States with trackable expansion over time. The results indicated that Omicron could have been detected 20 days earlier in Africa; similarly, 8 Omicron cases were detected in the United States by November 25, 2021, prior to the official US Centers for Disease Control and Prevention declaration.

**Meaning:**

These findings suggest that novel data analytics such as statistical learning strategy may have applications for surveillance of SARS-CoV-2 variants.

## Teaser

This cases series evaluates the application of a statistical learning strategy to existing surveillance data to improve the early detection of novel SARS-CoV-2 variants.

## Introduction

The COVID-19 Omicron variant has rapidly become a dominant variant in the United States following the first official report to the World Health Organization (WHO) on November 24, 2021, by South Africa and officially designated a variant of concern (VOC) on November 26, 2021, followed by the US Centers for Disease Control and Prevention (CDC) on November 30, 2021. Reports of infections, hospitalizations, and deaths in South Africa, the United Kingdom, and other countries^1,2^ suggest that Omicron is highly transmissible and has numerous breakthrough infections but causes relatively minor disease among vaccinated patients. Its rapid spread suggests that an earlier detection might help future planning for such highly transmissible variants. We present here a statistical learning strategy (SLS) for detecting new variants based on an established data resource, the Global Initiative on Sharing Avian Influenza Data (GISAID), which archives COVID-19 sequences worldwide.^3,4^ We applied this strategy to assess whether we could have detected the expansion of the Omicron variant earlier in Africa.

## Methods

### GISAID and Patient Populations

GISAID is a global science initiative and provides open-access to genomic data of SARS-CoV-2 in the COVID-19 pandemic.^3,4,5^ We accessed GISAID for full viral genome sequences collected from January 1, 2020, to December 28, 2021, from 63 686 patients in more than 30 African countries and used them to trace the origin of Omicron in Africa. Similarly, we retrieved 531 827 full viral genome sequences from the United States (October 1 to December 27, 2021) to track the expansion of the Omicron variant in all US states and territories. This study was determined to be exempt under 45 CFR § 46.104(d)(4), exempt from informed consent, and waived from further review by Fred Hutchinson Research Center institutional review board.

### Viral Sequences and Haplotypes

After obtaining sequences, we aligned them against the reference genome of the SARS-CoV-2 (Covid-ref-NC_045512) and performed quality control on aligned sequences. We then extracted nucleotides in the spike protein (21 563-25 483 base pair) and translated them into protein sequences. We identified 28 amino acid mutating substitutions, known as polymutants (PM) here, that constitute a core Omicron haplotype (A67V, T95I, G339D, S371L, S373P, S375F, K417N, N440K, G446S, S477N, T478K, E484A, Q493R, G496S, Q498R, N501Y, Y505H, T547K, D614G, H655Y, N679K, P681H, N764K, K796Y, N856K, Q954H, N69K, and L981F)^6^ because they were consistently observed among most Omicron genomes (L.P.Z., unpublished data, 2022). For missing PMs, we performed imputation to fill in missing residues (if posterior probabilities exceeded 95%) using observed polymorphic nucleotides (outside of the spike protein) and their haplotype structures. If imputation was not successful, remaining missing residues are denoted as *x* in sequences.

**Figure 1.**
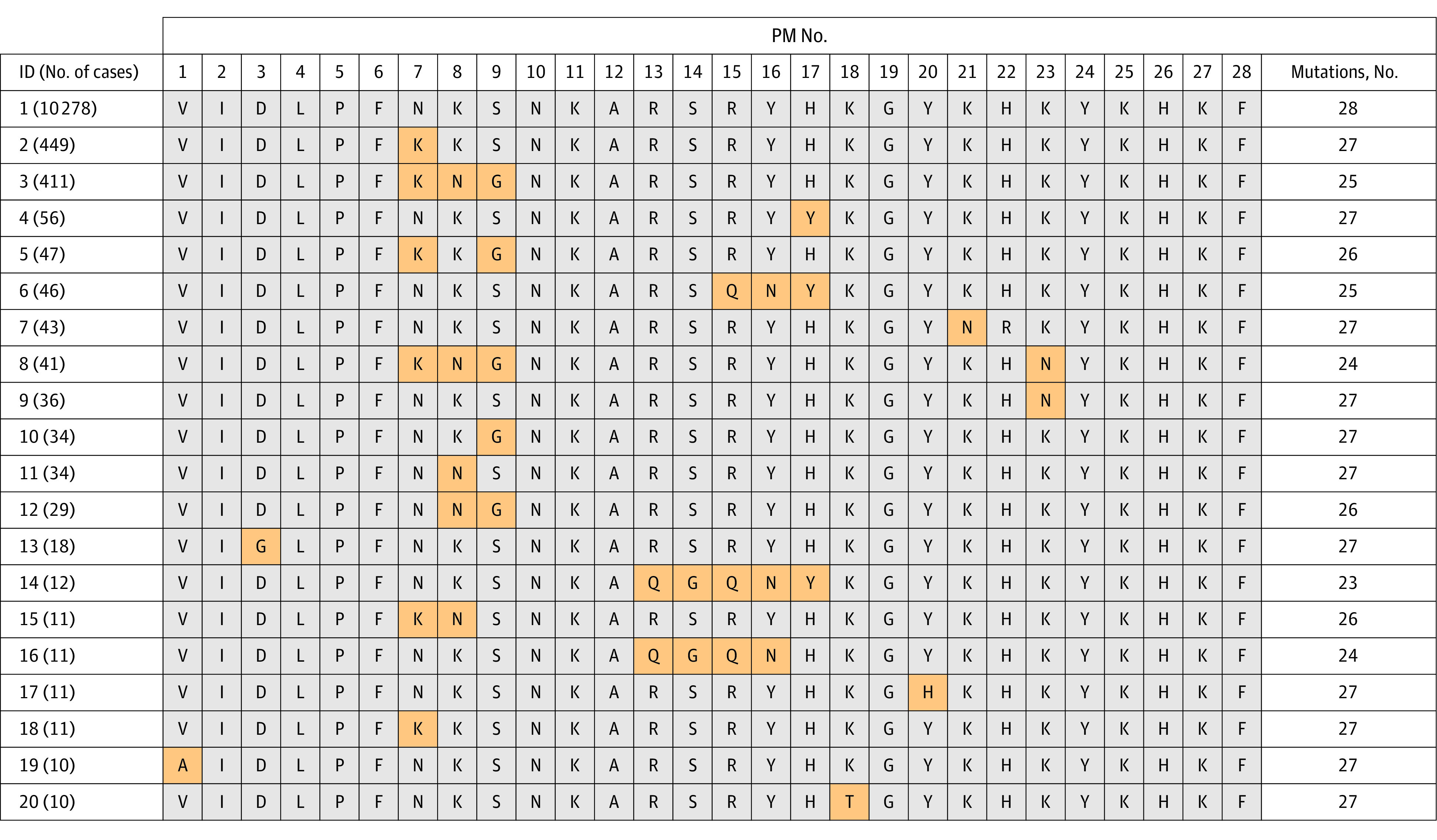
Omicron Haplotype and Polymorphic Variations in US Sequences Detected in 10 or More Viruses Omicron samples were collected from October 1 to December 24, 2021. Omicron haplotypes were assumed to have at least 10 mutations from the reference, and, in the event of missing amino acids, remaining amino acids have at least 80% of selected Omicron haplotypes. A full set of haplotypes in the United States are listed in eTable 1 in the Supplement, while those in Africa are listed in eTable 7 in the Supplement. A total of 28 polymutants (PMs) were selected, as follows: 1, A67; 2, T95; 3, G339; 4, S371; 5, S373; 6, S375; 7, K417; 8, N440; 9, G446; 10, S477; 11, T478; 12, E484; 13, Q493; 14, G496; 15, Q498; 16, N501; 17, Y505; 18, T547; 19, D614; 20, H655; 21, N679; 22, P681; 23, N764; 24, D796; 25, N856; 26, Q954; 27, N969; and 28, L981. ID indicates identifier.

Haplotypes of 28 Omicron mutations were used to define Omicron variants. We found that a core Omicron haplotype (VIDLPFNKSNKARSRYHKGYKHKYKHKF) characterizes 90% of all Omicron viruses (L.P.Z., unpublished data, 2022). However, this haplotype remained polymorphic, with newly emerging mutations or missing residues. To balance sensitivity and specificity, we set up a base set of Omicron haplotypes and then expanded the base set to include additional haplotypes if the maximum sequence similarity to any initial base haplotype exceeded 80% with 4 or fewer missing observations, or a maximum sequence similarity greater than 90% if there were more than 4 missing observations. As a result, we identified a total of 343 polymorphic haplotypes, each of which had from 10 to 28 mutations in the spike protein relative to the original SARS-CoV-2 sequence. Figure 1 presents 20 haplotypes observed 10 or more times, and eTable 1 in the Supplement includes a complete list. Among 12 141 Omicron viruses identified in US using the haplotype protocol, 98% of them were of the BA.1 (n = 11 900) or B.1.1.529 (n = 44) lineages assigned by Phylogenetic Assignment of Named Global Outbreak Lineages (PANGO) software^7^ (eTable 2 in the Supplement). We additionally identified 1 case of BA.2, 2 cases of BA.3, 4 cases of B.1, 2 cases of B.1.1., 1 case each of B.1.1.161 and B.1.1.523, and 1 case each of AY.1, AY.103, AY.42 lineages. The remaining 183 cases were unclassified. The primary reason for choosing this haplotype protocol to identify the Omicron cases, rather than using the PANGO assigned lineages, is that this strategy can rapidly identify new variants before any lineage is established and assigned, as explained in the Discussion section.

### Outcome

We used the set of 343 Omicron haplotypes to identify Omicron viruses. In total, there were 2698 Omicron cases in Africa (eTable 3 in the Supplement) and 12 141 Omicron cases in the United States (eTable 4 in the Supplement).

### Sample Collection Time and Location

Metadata associated with each viral sequence included sample collection date and location. Their distributions in Africa are listed in the eTable 3 in the Supplement, and those in the United States in eTable 4 in the Supplement. The location was organized by continent, country, region, and subregion. Metadata also included assigned clade by GISAID^5^ and lineage by PANGO.^7^

### Statistical Analysis

To analyze viral sequences, we applied an SLS, including several analytic approaches,^8^ including modeling Omicron temporal expansion over time,^8,9^ an unsupervised learning technique,^10^ and a haplotype-based imputation method and bootstrapping confidence intervals. Technical details are described in the eAppendix in the Supplement. All statistical analyses were performed with functions in R version 4.0.5 (R Project for Statistical Computing). In temporal analyses, a PM was selected if *P* for nonlinear trend was less than .05.

## Results

### Omicron Haplotypes

Most Omicron viruses shared 28 mutations in the spike protein (L.P.Z., 2022, unpublished data). Their haplotypes were polymorphic, with a single dominant haplotype VIDLPFNKSNKARSRYHKGYKHKYKHKF in the United States (Figure 1), which accounted 85% of all Omicron viruses. The second most common haplotypes deviated from the dominant haplotype by K417K, while the third deviated by N440N and G446G. Among these Omicron haplotypes, they had at least 23 mutating sites indicated by the value in the last column of Figure 1. In the expanded set of 343 haplotypes (eTable 1 in the Supplement), the Omicron haplotype had variable numbers of mutations (at least 10 mutations as the threshold).

### Geospatial and Temporal Distributions of Omicron Viruses in Africa

To investigate geospatial and temporal distributions of the Omicron variant in Africa, we focused on Omicron-positive cases and tabulated their collection dates within countries (eTable 5 in the Supplement). There was a single Omicron case collected on December 31, 2020, in South Africa, and all other Omicron cases were collected during 2021 (eTable 5 in the Supplement). It is noteworthy that the first Omicron sample was collected on December 31, 2020, but only 12 residues in the haplotype were mutated (Table 1). Nearly 10 months later, South Africa began experiencing an escalation of Omicron cases, starting on September 30, 2021. Shortly afterwards, Nigeria identified its first Omicron case on October 17, 2021; Senegal its first case on November 9, 2021; and Botswana its first case on November 11, 2021.

**Table 1.** First Omicron Cases Detected in Africa and the United States

ID	Collection date	Location	Gender	Age	Lineage	Clade	Spike haplotype	Mutations, No.
**First 13 cases in Africa**								
1	12/31/2020	South Africa, Eastern Cape	Male	57	B.1.576	GRA	VIGSSSNXGSTKQGQYYXGYKHXDXHKF	12
2	9/30/2021	South Africa, Gauteng	Female	52	BA.1	G	VIDLPFNKSNKARSRYHKGYKHKYKHKF	28
3	10/12/2021	South Africa, Eastern Cape	Female	16	BA.1	GRA	VIDLPFNKSSKEQGQYYKGYKHKYKHKF	22
4	10/17/2021	Nigeria, Abuja	Male	32	BA.1	GRA	VIGSSSKKSNKARSRYHKGYKHKYKHKF	23
5	10/24/2021	South Africa, Eastern Cape	Female	22	BA.1	GRA	VIDLPFNKSNKARSRYHKGYKHKYKHKF	28
6	11/2/2021	Nigeria, Abuja	Male	51	BA.1	GRA	VIXXXXXXXXXXXXXXXKGYKHKYNHKF	12
7	11/2/2021	South Africa, Northern Cape	Female	28	BA.1	GRA	VIDLPFNKSNKARSRYHKGYKHKYKHKF	28
8	11/5/2021	South Africa, Gauteng	Male	26	BA.1	GRA	VIDLPFNKSNKARSRYHKGYKHKYKHKF	28
9	11/8/2021	South Africa, Gauteng	Unknown	Unknown	BA.1	GRA	VIDLPFNKSNKARSRYHKGYKHKYKHKF	28
10	11/9/2021	South Africa, Gauteng	Male	23	BA.1	GRA	VIDLPFNKSNKARSRYHKGYKHKYKHKF	28
11	11/9/2021	South Africa, Gauteng	Male	34	BA.1	GRA	VIDLPFNKSNKARSRYHKGYKHKYKHKF	28
12	11/9/2021	South Africa, Gauteng	Unknown	Unknown	BA.1	GRA	VIDLPFNKSNKARSRYHKGYKHKYKHKF	28
13	11/9/2021	Senegal/Dakar, Iressef Diamniadio	Female	42	B.1.1.529	G	VIDLPFKKSNKARSRYHKGYKHKYKHKF	27
**First 8 cases in the United States**								
1	11/21/2021	Maryland	Female	40	BA.1	GRA	VIDLPFNKSNKARSRYHKGYKHKYKHKF^a^	28
2	11/22/2021	New York City	Male	33	BA.1	GRA	VIDLPFNKSNKARSRYHKGYKHKYKHKF	28
3	11/24/2021	Minnesota	Unknown	Unknown	BA.1	GRA	VIDLPFNKSNKARSRYHKGYKHKYKHKF	28
4	11/24/2021	New York City	Female	32	BA.1	GRA	VIDLPFNKSNKARSRYHKGYKHKYKHKF	28
5	11/24/2021	Missouri	Female	25	BA.1	GRA	VIDLPFNKSNKARSRYHKGYKHKYKHKF	28
6	11/24/2021	Virginia	Female	23	BA.1	GRA	VIDLPFNKSNKARSRYHKGYKHKYKHKF	28
7	11/25/2021	New York City	Male	30	BA.1	GRA	VIDLPFNKSNKARSRYHKGYKHKYKHKF	28
8	11/25/2021	New York	Male	26	BA.1	GRA	VIDLPFNKSNKARSRYHKGYKHKYKHKF	28

Abbreviation: ID, identification.

^a^ This Omicron haplotype is imputed to be the same core haplotype from VIDXXXXXXXXXXXXXXKGYKHKYKHKF, in which multiple missing residues were consecutive and were likely due to sequencing quality.

### First 13 Omicron Cases in Africa

Given interest in the origin of the Omicron variant, we listed the first 13 cases detected by November 9, 2021 (Table 1). Ten samples were collected in South Africa, 2 from Nigeria, and 1 from Senegal. Both male and female patients were included. Their ages ranged from 22 to 57 years. Their haplotypes included at least 12 mutated amino acids, as indicated in the last column. As noted previously, the first example of the extreme mutation profile characteristic of the Omicron variant was reported in South Africa on December 31, 2020. These early case reports were from Eastern Cape, South Africa (Table 1 and eTable 6 in the Supplement). Case 1, with 12 mutations, and case 3, with 22 mutations, shared 11 mutations, and case 2 shared 22 mutations with the core Omicron haplotype. Both temporal and geographic factors suggest that these 3 cases may be related, and this may merit further investigation.

### Temporal Trends and Detection Times in Africa

**Figure 2.**
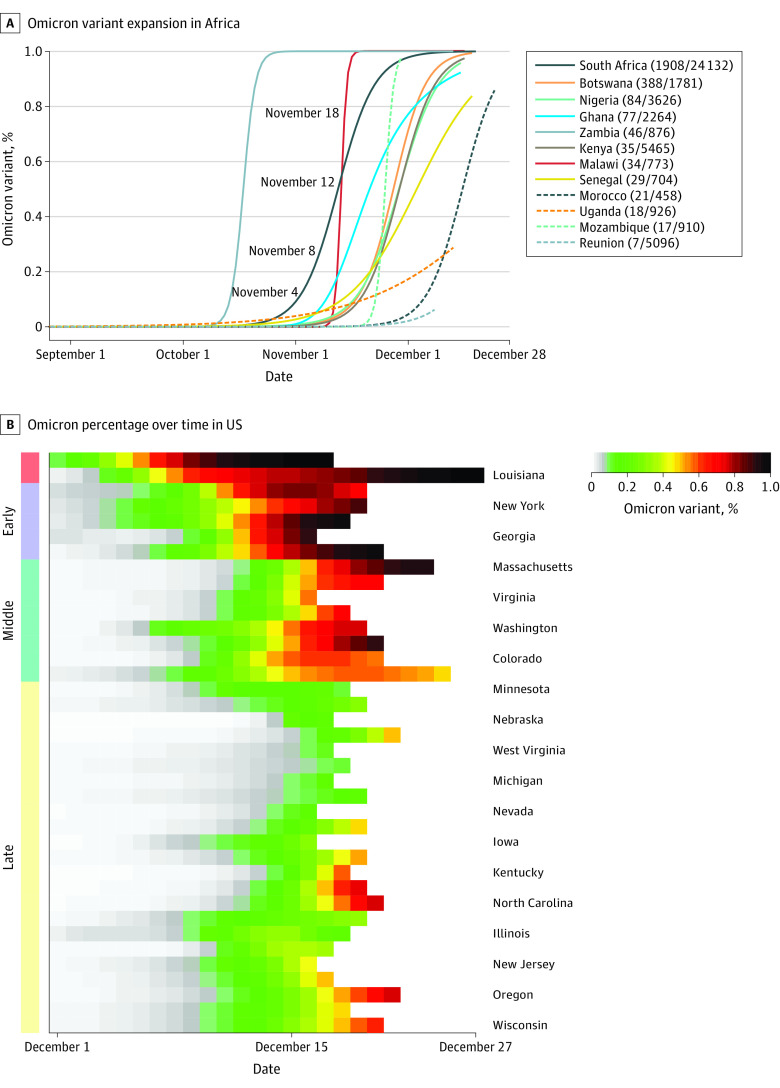
Locally Averaged Omicron Caseload Percentages A, Omicron variant expansion in African countries. B, Omicron percentages over time across US states, organized by temporal pattern similarities.

We proceeded to model the temporal trends of Omicron occurrence in 12 African countries that deposited 5 or more Omicron genomes (Figure 2A). All countries had significant temporal trends (data not shown). Omicron caseload percentages (OCPs) were computed from January 1, 2020, to December 28, 2021. Most African countries had fewer than 100 Omicron cases during this surveillance period, and Botswana exhibited emergence of Omicron later than South Africa. These considerations led us to use South Africa as a benchmark to evaluate the increase in Omicron caseload over time and the progression time required for the OCP to reach 10%, 25%, 50%, or 75% of total coronavirus cases (Table 2). Since the initial Omicron case identification on December 31, 2020, the OCP reached 10% by November 4 (95% CI, October 31 to November 6), 2021, or in 306 days (95% CI, 302 to 308) from first Omicron sequence identification. Similarly, the OCP reached 25%, 50%, and 75%, respectively, by day 311 (95% CI, 309 to 313), 317 (95% CI, 315 to 318) and 322 (95% CI, 321 to 323) from initial identification date, corresponding to November 8, 12, and 18, 2021. The emergence patterns in other countries were similar and their estimated days to reach respective threshold percentages were computed (Figure2A and Table 2). Note that estimated detection timings in several countries, such as Zambia, were negative, due to a diverse expansion pattern (eFigure 1 in the Supplement) that led to negative values (eFigure 2 in the Supplement).

**Table 2.** Estimated Time for Omicron Caseload Percentages to Reach the 10%, 25%, 50%, and 75% Thresholds Across 12 African Countries

African country	Frequency^a^		First detection	Time to targeted percentage, d (95% CI)^b^			
	Negative	Positive		10%	25%	50%	75%
South Africa	24 132	1908	12/31/2020	306 (302 to 308)	311 (309 to 313)	317 (315 to 318)	322 (321 to 323)
Nigeria	3626	84	10/17/2021	31 (25 to 38)	37 (34 to 41)	43 (41 to 45)	49 (46 to 51)
Senegal	704	29	11/9/2021	5 (−6 to 19)	15 (7 to 24)	25 (17 to 31)	35 (27 to ∞)
Ghana	2264	77	11/10/2021	0 (−4 to 5)	5 (1 to 9)	11 (7 to 15)	20 (16 to ∞)
Botswana	1781	388	11/11/2021	7 (−1 to 9)	12 (6 to 14)	17 (15 to 19)	21 (20 to 23)
Reunion	5096	7	11/22/2021	NA	NA	NA	NA
Kenya	5465	35	11/26/2021	−7 (−10 to −4)	−2 (−4 to 1)	3 (0 to 7)	8 (4 to 14)
Mozambique	910	17	11/26/2021	−3 (−21 to 2)	−2 (−20 to 2)	−1 (−20 to 2)	1 (−19 to 3)
Uganda	926	18	11/29/2021	−9 (−18 to 3)	11 (2 to ∞)	NA	NA
Zambia	876	46	11/30/2021	−46 (−49 to −45)	−44 (−47 to −43)	−42 (−45 to −41)	−41 (−43 to −39)
Malawi	773	34	12/2/2021	−20 (−41 to −19)	−19 (−40 to −18)	−18 (−38 to −18)	−18 (−36 to −17)
Morocco	458	21	12/14/2021	−8 (−15 to −2)	−3 (−8 to 1)	2 (−2 to 6)	7 (3 to ∞)

Abbreviation: NA, not applicable.

^a^ Frequencies of viruses carrying Omicron (positive) or not (negative).

^b^ The 95% CIs were obtained from 2.5% and 97.5% of the empirically computed distribution from 1000 bootstraps. The time was inestimable if the Omicron caseload had not crossed the target percentage. The upper bound of the 95% CI was left as ∞ if it was beyond December 28, 2021. A negative day is an extrapolated value from the model fitting to sparse data that the Omicron percentage jumped from 0% to 100% over a short time (see example in eFigures 1 and 2 in the Supplement on Zambia).

### First 8 Omicron Cases in the United States Identified Prior to November 26, 2021

At the time of declaring Omicron a VOC by the CDC on November 30, 2021, there were no officially reported Omicron cases in the United States. Eight Omicron samples were collected between November 21 and 25, 2021, and were sequenced according to the collection dates recorded in the GISAID (Table 1). Age ranges of these cases were from 25 to 40 years, including both male and female patients. They were from Maryland, New York City, Virginia, New York State, Minnesota, and Missouri. They all shared the same core Omicron haplotype.

### OCP in the United States

Excluding states or territories with fewer than 10 Omicron cases, we computed OCP from October 1 to December 27, 2021, in 38 states and organized their Omicron expansions by their temporal similarities. Since few Omicron cases were identified in November 2021, Figure, B, showed the Omicron caseload percentage from December 1 to 27, 2021. There were no other Omicron cases identified prior to November 26, 2021, except for those 8 cases discussed previously (Table 2). Once an Omicron case was identified in each state, its percentage of all COVID-19 cases rose rapidly. Seven states and territories (Hawaii, Louisiana, Texas, New York, District of Columbia, Georgia, and Florida) were clustered into an early group. The next cluster of 8 states represented the next Omicron wave, the middle group (Figure, B), while the remaining 23 states had not yet experienced the spread of the Omicron variant by December 27. To quantify the spread of the Omicron variant on individual states, we computed the number of days for OCP to reach 25%, 50%, or 75%, following the first case report within that state (Table 3). Hawaii, one of the first states to experience Omicron, documented its first Omicron case on November 27, and the OCP rose to 25% within 7 days (95% CI, 6-8 days), then reached 50% and 75% within 9 days (95% CI, 8-10 days) and 11 days (95% CI, 10-13 days), respectively. Washington State, in the middle phase cluster, reported its first Omicron case on November 27, and its OCP rose to 25% and 50% within 15 days (95% CI, 14-15 days) and 18 days (95% CI, 18-19 days), respectively. Oregon, one of the last states to document Omicron cases, reported its first case on December 7, and its OCP increased to 25% and 50% within 8 days (95% CI, 7-9 days) and 11 days (95% CI, 10-13 days), respectively. The overall trend for Omicron progression in the United States is similar to that observed in South Africa and most other African nations, but temporal patterns vary notably from state to state.

**Table 3.** Estimated Time for Omicron Caseload Percentages to Reach the 10%, 25%, 50%, and 75% Thresholds Across 38 US States

States	Frequency^a^		First detection	Time to targeted percentage, d (95% CI)^b^			Cluster group
	Negative	Positive		25%	50%	75%	
Alabama	930	15	12/5/2021	9 (−1 to 11)	12 (10 to ∞)	NA	Middle
Arizona	15 452	168	12/5/2021	11 (11 to 12)	14 (14 to ∞)	NA	Late
California	78 404	3426	11/26/2021	18 (18 to 18)	20 (20 to 21)	NA	Middle
Colorado	49 426	396	11/29/2021	13 (12 to 13)	15 (14 to 16)	NA	Middle
Connecticut	8596	162	11/28/2021	18 (16 to ∞)	NA	NA	Late
DC	1697	307	11/30/2021	10 (9 to 11)	12 (11 to 13)	14 (14 to 15)	Early
Florida	9690	275	11/26/2021	14 (14 to 15)	17 (16 to 17)	19 (19 to 21)	Early
Georgia	4687	274	11/30/2021	11 (10 to 12)	13 (12 to 13)	14 (14 to 15)	Early
Hawaii	1364	93	11/27/2021	7 (6 to 8)	9 (8 to 10)	11 (10 to 13)	Early
Idaho	3166	21	12/5/2021	12 (10 to 14)	NA	NA	Late
Illinois	13 425	248	11/30/2021	12 (12 to 14)	NA	NA	Late
Indiana	11 120	93	12/8/2021	8 (7 to 8)	10 (9 to 11)	NA	Late
Iowa	4365	31	12/6/2021	10 (8 to ∞)	NA	NA	Late
Kansas	4289	20	12/13/2021	6 (5 to ∞)	8 (7 to ∞)	NA	Late
Kentucky	4168	38	12/11/2021	5 (4 to 6)	7 (6 to ∞)	NA	Late
Louisiana	1742	333	12/1/2021	5 (4 to 6)	7 (6 to 8)	12 (9 to 16)	Early
Maryland	8982	395	11/21/2021	21 (20 to 21)	25 (24 to 26)	NA	Middle
Massachusetts	40 270	288	11/27/2021	18 (17 to 18)	19 (19 to 20)	22 (21 to ∞)	Middle
Michigan	19 568	21	12/1/2021	NA	NA	NA	Late
Minnesota	34 004	76	11/24/2021	NA	NA	NA	Late
Mississippi	1442	13	11/29/2021	20 (15 to ∞)	NA	NA	Late
Nebraska	5447	21	11/29/2021	NA	NA	NA	Late
Nevada	6691	23	12/8/2021	NA	NA	NA	Late
New Jersey	11 479	203	11/26/2021	18 (16 to 19)	NA	NA	Late
New York	23 536	1975	11/22/2021	17 (17 to 18)	21 (21 to 22)	25 (24 to 25)	Early
North Carolina	10 833	145	12/2/2021	14 (13 to 14)	16 (15 to 17)	18 (17 to ∞)	Late
Ohio	9686	376	11/29/2021	14 (13 to 14)	16 (16 to 17)	19 (18 to 20)	Middle
Oregon	4935	83	12/7/2021	8 (7 to 9)	11 (10 to 13)	NA	Late
Pennsylvania	13 210	96	11/28/2021	15 (15 to 17)	NA	NA	Late
Rhode Island	3507	15	11/30/2021	NA	NA	NA	Late
South Carolina	2732	56	12/4/2021	11 (9 to 12)	NA	NA	Late
Tennessee	4602	35	11/26/2021	19 (18 to 21)	NA	NA	Late
Texas	16 336	1096	11/27/2021	12 (12 to 13)	14 (14 to 15)	17 (16 to 17)	Early
Utah	9338	20	11/29/2021	NA	NA	NA	Late
Virginia	5088	94	11/24/2021	20 (19 to 21)	22 (21 to ∞)	NA	Middle
Washington	13 643	688	11/27/2021	15 (14 to 15)	18 (18 to 19)	NA	Middle
West Virginia	6415	19	12/2/2021	NA	NA	NA	Late
Wisconsin	13 477	435	11/27/2021	18 (18 to 18)	22 (21 to 22)	NA	Late

Abbreviation: NA, not applicable.

^a^ Frequencies of viruses carrying Omicron (positive) or not (negative).

^b^ The 95% CIs were obtained from 2.5% and 97.5% of the empirically computed distribution from 1000 bootstraps. The time was inestimable if the Omicron caseload had not crossed the targeted percentage. The upper bound of the 95% CI was left as ∞ if it was beyond December 28, 2021.

## Discussion

This case series using viral sequences from African countries and the United States suggests that the first Omicron sequence was identified in South Africa on December 31, 2020. The OCP in South Africa reached 10%, 25%, 50%, and 75% by November 4, 8, 12, and 18, 2021, respectively. While the first Omicron progenitor, with 12 Omicron mutations, was collected on December 31, 2020, and was unlikely to be informative of future expansion, it should not be treated as the first date of detecting the Omicron variant. Instead, we should treat the second identified sequence, with 22 Omicron mutations, as a potential progenitor (Figure2A). By November 4, 2021, the OCP reached 10%, and this threshold was suggested as an empirical threshold for a public health alert.^8^ Our retrospective study illustrates that South Africa and other African nations collected valuable sequence data that could have been used to track the emergence of Omicron variants, providing potentially useful information as many as 22 days prior to the officially declaration on November 26, 2021.

Earlier detection of the Omicron variant and documentation of its rapid transmission might have been of great benefit. Public health policies may have been implemented to limit or delay rapid global transmission, and clinicians would have had more advance warning of yet another caseload surge. Since structural information is available for several SARS-CoV-2 proteins, particularly the spike protein, early information regarding key mutations could be used in protein homology modeling studies to anticipate potential effects on vaccine or therapeutic antibody effectiveness. For example, the Omicron variants carry mutations in spike protein regions that are known binding sites for some current therapeutic antibodies, and earlier information that the effectiveness of these therapeutic antibodies against Omicron variants might be compromised would be valuable for both clinicians and scientists involved in antibody development.

In the current study, we chose to use a set of Omicron haplotypes to define the Omicron virus as opposed to conventional lineages assigned by PANGO.^7^ Indeed, use of conventionally designated lineages for this retrospective study is straightforward given that all viral sequences are automatically assigned lineages by GISAID when sequences are submitted. The phylogenic approach accounts for both nucleotide mutations and insertions and deletions. However, the rapid mutation rate observed for coronaviruses can make it challenging to revise and update lineage designations in a timely manner. In contrast, the SLS,^8^ relying on PM, identifies new mutation haplotypes with as few as 1 to 3 samples if the haplotype includes, for example, 10 or more mutated residues. Essentially, the haplotype approach enables the discovery of new variants without designated lineages. For example, the first omicron virus was assigned the lineage B.1.576, rather than BA.1. Additionally, when haplotype-tagged variants become dominant, this approach could facilitate revising lineages and designating appropriate variants. Despite its advantages, SLS has benefited from assigned lineages in identifying Omicron core haplotypes and is best viewed as a complementary approach to the phylogenic analysis at this time.

Metadata in the GISAID database includes several fields for disease severity or vaccination status. If such data were routinely submitted, it would facilitate correlation of new variants with clinical metrics, such as our recent discovery of a viral haplotype that associates with hospitalization risk.^11^ Unfortunately, data submissions at this point are full of missing and/or incoherent values and remain to be improved.

An interesting and potentially important observation is that there were 3 early Omicron cases in Eastern Cape, South Africa (case identifications 1, 3, and 5) with 12, 22, and 28 mutations, respectively, in a relatively short period of time (Table 1; eTable 6 in the Supplement). This temporal clustering implies that the original isolate may have been transmitted and that the infected person may have accumulated additional mutations with limited intermediaries. This observation appears to suggest that there may have been micro-outbreaks in a community of immunocompromised persons who are at risk of prolonged persistent infection, instead of originating in 1 or 2 persons.

### Limitations

This study has several limitations. Despite a large collection of samples, it was an observational study, and results are descriptive in nature. Given the nature of volunteer submission to GISAID, we have limited information on sampling population, ie, the denominator, and hence are unable to estimate disease prevalence or incidence rates. Instead, what can be reliably estimated are percentages as OCP, which are nevertheless indicative of the prevalence or incidence of patients with the Omicron variant of COVID-19. Another limitation is that viral sequences in several African countries were not collected continuously, and sparse data collection necessitates extrapolations that may rely too much on assumptions in the generalized additive model. Hence, estimated days that OCP reached a designated percentage need to be interpreted with caution. The third limitation is that the Omicron haplotypes used only 28 mutations in the spike protein. When deploying the strategy, it may be necessary to monitor all PM in all viral genes in addition to the spike protein and also to consider synonymous nucleotide substitutions in or outside of genes; although synonymous mutations yield an unchanged protein sequence, previous experimental studies have shown that synonymous mutations can impact ribosomal translation kinetics, which may lead to altered protein folding in some cases.^12,13,14^ Whether due to altered protein folding or simply enhanced protein production via accelerated ribosomal translation, synonymous mutations may increase what is known as viral fitness, leading to viral variants with clinical significance. The fourth limitation is that submission date may be delayed by days or weeks after collection date.^15^ Any significant lag time between data collection and submission will impede our ability to detect new viral variants in a timely manner, so it may be important to take steps as a community to improve data generation and submission efficiency. The fifth limitation is that determining OCP benefited from fitted temporal OCP curves and relied on all observations. Therefore, estimated timing may be thought of as a best-case scenario when applying this method for detecting future variants.

## Conclusions

This study suggests that given the amount and quality of sequence data available from South Africa and other African countries, it may have been possible to detect initial Omicron cases earlier than reported. Building on GISAID, SLS with or without assigned lineages may be used to identify emerging variants.

## Notes

Supplement. **eAppendix. **Statistical Learning Strategy **eTable 1.** Omicron Haplotype and Its Polymorphic Variations in the United States, October 1 to December 27, 2021 **eTable 2.** Distribution of Lineages Among Omicron Cases in Africa and the United States **eTable 3.** Frequency Distributions of Gender, Collection Date, and Collection Countries Among Non-Omicron Cases and Omicron Cases in This Study, With Positive Percentage Computed, Among a Total of 63 686 COVID-19 Cases From African Countries **eTable 4.** Frequency Distributions of Gender, Collection Date, and Collection Countries Among Non-Omicron Cases and Omicron Cases in This Study, With Positive Percentage Computed, Among a Total of 531 827 COVID-19 Cases From the United States **eTable 5.** Frequencies of Observed Omicron viruses in African Countries: Botswana, Egypt, Ghana, Kenya, Malawi, Morocco, Mozambique, Nigeria, Reunion, Senegal, South Africa, Uganda, Zambia, and Other Countries From October 31, 2020, to December 28, 2021 **eTable 6.** First 3 Omicron Cases From Eastern Cape, South Africa **eTable 7.** Omicron Haplotype and Its Polymorphic Variations in African Countries, January 1, 2020, to December 28, 2021 **eFigure 1.** Heatmap Representation of Polymutant Expansions in Zambia **eFigure 2.** Illustration on Negative Estimate of Detection Time Due to Sparse Collection of Viral Sequences During a Critical Period When a Polymutant Rises Rapidly to Become a Dominant Mutation in a Short Period of Time
eReferences.
