# Error in Figure 2

**DOI:** 10.1001/jamanetworkopen.2022.39195

**Published:** 2022-10-06

In the Original Investigation titled “Application of Statistical Learning to Identify Omicron Mutations in SARS-CoV-2 Viral Genome Sequence Data From Populations in Africa and the United States,”^1^ published September 7, 2022, there was an error in Figure 2. In panel B, the list of states was incomplete. This article has been corrected.^1^

## References

[1] Zhao LP, Lybrand TP, Gilbert P, . Application of statistical learning to identify Omicron mutations in SARS-CoV-2 viral genome sequence data from populations in Africa and the United States. JAMA Netw Open. 2022;5(9):e2230293. doi:10.1001/jamanetworkopen.2022.30293 36069983 PMC9453543

