# Supplementary material for: Application of Statistical Learning to Identify Omicron Mutations in SARS-CoV-2 Viral Genome Sequence Data From Populations in Africa and the United States
Source: JAMA Netw Open. 2022 Sep 7;5(9):e2230293. doi: 10.1001/jamanetworkopen.2022.30293 (PMC9453543; doi:10.1001/jamanetworkopen.2022.30293)
Supplement: Supplement. — eAppendix. Statistical Learning Strategy eTable 1. Omicron Haplotype and Its Polymorphic Variations in the United States, October 1 to December 27, 2021 eTable 2. Distribution of Lineages Among Omicron Cases in Africa and the United States eTable 3. Frequency Distributions of Gender, Collection Date, and Collection Countries Among Non-Omicron Cases and Omicron Cases in This Study, With Positive Percentage Computed, Among a Total of 63 686 COVID-19 Cases From African Countries eTable 4. Frequency Distributions of Gender, Collection Date, and Collection Countries Among Non-Omicron Cases and Omicron Cases in This Study, With Positive Percentage Computed, Among a Total of 531 827 COVID-19 Cases From the United States eTable 5. Frequencies of Observed Omicron viruses in African Countries: Botswana, Egypt, Ghana, Kenya, Malawi, Morocco, Mozambique, Nigeria, Reunion, Senegal, South Africa, Uganda, Zambia, and Other Countries From October 31, 2020, to December 28, 2021 eTable 6. First 3 Omicron Cases From Eastern Cape, South Africa eTable 7. Omicron Haplotype and Its Polymorphic Variations in African Countries, January 1, 2020, to December 28, 2021 eFigure 1. Heatmap Representation of Polymutant Expansions in Zambia eFigure 2. Illustration on Negative Estimate of Detection Time Due to Sparse Collection of Viral Sequences During a Critical Period When a Polymutant Rises Rapidly to Become a Dominant Mutation in a Short Period of Time eReferences. [file jamanetwopen-e2230293-s001.pdf]

Supplemental Online Content

Zhao LP, Lybrand TP, Gilbert P, et al. Application of statistical learning to identify Omicron mutations in SARS-CoV-2 viral genome sequence data from populations in Africa and the United States. *JAMA Netw Open.* 2022;5(9):e2230293. doi:10.1001/jamanetworkopen.2022.30293

eAppendix. Statistical Learning Strategy

eTable 1. Omicron Haplotype and Its Polymorphic Variations in the United States, October 1 to December 27, 2021

eTable 2. Distribution of Lineages Among Omicron Cases in Africa and the United States

eTable 3. Frequency Distributions of Gender, Collection Date, and Collection Countries Among Non-Omicron Cases and Omicron Cases in This Study, With Positive Percentage Computed, Among a Total of 63 686 COVID-19 Cases From African Countries

eTable 4. Frequency Distributions of Gender, Collection Date, and Collection Countries Among Non-Omicron Cases and Omicron Cases in This Study, With Positive Percentage Computed, Among a Total of 531 827 COVID-19 Cases From the United States

eTable 5. Frequencies of Observed Omicron viruses in African Countries: Botswana, Egypt, Ghana, Kenya, Malawi, Morocco, Mozambique, Nigeria, Reunion, Senegal, South Africa, Uganda, Zambia and Other Countries From October 31, 2020, to December 28, 2021

eTable 6. First 3 Omicron Cases From Eastern Cape, South Africa

eTable 7. Omicron Haplotype and Its Polymorphic Variations in African Countries, January 1, 2020, to December 28, 2021

eFigure 1. Heatmap Representation of Polymutant Expansions in Zambia

eFigure 2. Illustration on Negative Estimate of Detection Time Due to Sparse Collection of Viral Sequences During a Critical Period When a Polymutant Rises Rapidly to Become a Dominant Mutation in a Short Period of Time

eReferences.

This supplemental material has been provided by the authors to give readers additional information about their work.

© 2022 Zhao LP et al. *JAMA Network Open.*

eAppendix. Statistical Learning Strategy

Statistical Learning Strategy (SLS) includes a set of statistical and computation methods for processing and analyzing viral sequence data.

1. Sequence data and alignment

SARS-COV-2 viral sequences from COVID-19 cases in Africa and the US, along with their associated metadata, are retrieved from GISAID (https://www.gisaid.org accessed on December 28, 2022). Nucleotides in the spike protein (from 21563 BP to 25384 BP) are extracted and are aligned to the Wuhan reference sequence (Covid-ref-NC_045512). Applying the alignment tool MAFFT [1,2] leads to aligned nucleotide sequences, which can then be translated to amino acid (AA) sequences in the spike protein. Selecting 28 Omicron polymutants (PM) leads to a rectangular-shaped PM matrix, denoted as $PM=|pm|_{Nx28}$ for N viral sequences, following the imputation (see the next section 2). In comparison with 28 PM in the reference sequence, one can also construct a matrix of binary indicators (0, 1) for presence or absence of substituting mutations, respectively. Let $Y=|y|_{Nx28}$ denote a matrix of binary indicators.

2. Imputing missed PM observations

Some of PM observations may be missing, due to missing nucleotides or structural insertions/deletions in the original sequences. Such missing observations could inflate polymorphisms of PM haplotypes. To minimize haplotypic polymorphisms, we impute missing PM observations, with DNA nucleotides. Since SARS-COV-2 is a single-stranded RNA sequence and most nucleotides are in high linkage disequilibrium, the haplotype structure allows us to impute missing PM, in a similar way as imputing HLA genotypes by single nucleotide polymorphisms in human genetics[3,4]. We construct a "imputation set of 10~100 nucleotides", say $(n_1, n_2, \ldots, n_{100})$, that have few missing values and are physically adjacent to spike protein. For each PM with missing observations, we perform haplotype analysis and estimate their empirical haplotype frequencies. Treating haplotype frequencies as the joint distribution function of the PM and selected nucleotides, denoted as $f(pm, n_1, n_2, \ldots, n_{100})$, we can deduce a posterior probability for PM observation given selected nucleotides

© 2022 Zhao LP et al. *JAMA Network Open.*

$$\Pr(pm \mid n_1, n_2, ..., n_{100}) = f(pm, n_1, n_2, ..., n_{100}) / \sum_{AA} f(pm = AA, n_1, n_2, ..., n_{100}), \qquad [1]$$

in which the summation is over all possible amino acids. For a PM with missing value, we compute the above posterior probability given observed nucleotides $(n_1, n_2, ..., n_{100})$. If a posterior probability of a particular amino acid exceeds a preset threshold value, say 99%, the corresponding amino acid is treated as the imputed PM value. In case that none of posterior probabilities exceed the preset threshold value, the PM observation is kept as a missing residue and is denoted by "X".

3. Defining Omicron variant

We define a virus as an Omicron variant if its haplotype of 28 PM has ten or more substituting mutations. Since some PM have missing values, we call out a Omicron variant, if its viral sequence, excluding missing observations, is identical to one of Omicron viral sequences with at least 80% PM identical. Let $y_i = 1 \text{ or } 0$ indicate that the *i*th virus is an Omicron variant or not, respectively.

4. Modeling Omicron expansion and estimating detection times

As expected, the Omicron viruses expand over time. To model the non-linear temporal expansion, we apply a generalized additive model (GAM) to regress the Omicron variant indicator $Y = (y_1, y_2, ..., y_N)$ over sample collection time via the following probability model

$$\Pr(y_i = 1 \mid t_i) = \frac{1}{1 + \exp[-\alpha - s(t_i)]} \qquad [2]$$

where α is a constant coefficient and $s(t_i)$ is a non-linear smooth function of time t_i, and both are estimated by the restricted maximum likelihood method [5]. Upon completing the estimation, the above function was used with the estimated coefficient and non-linear function to compute locally averaged Omicron caseload percentage (OCP). The function "gam" was used to fit the GAM (R packages MGCV). Default smoothing parameter is chosen. Upon fitting the GAM, the fitted values were used as locally averaged OCP daily from the first to the last reporting day. Computed proportions over time describe temporal expansion.

© 2022 Zhao LP et al. *JAMA Network Open*.

Based on the fitted model with estimated parameter $\hat{\alpha}$ and smooth function $\hat{s}(t_i)$, we can now estimate the timing that the OCP crosses a specific OCP value, i.e., detection time of OCP, via

$$t_{OCP} = \hat{s}^{-1}[\text{logit}(\text{OCP}) - \hat{\alpha}], \quad [3]$$

in which $\hat{s}^{-1}(.)$ is an inversed function of the smoothed function $\hat{s}(t_i)$, and logit(.) is the logit function. Since $\hat{s}(t_i)$ is non-parametrically estimated, we compute the detection time up to a specific day when the corresponding probability is the closest to the target OCP.

5. Bootstraping confidence interval for estimated detection time

Due to nature of non-parametrically estimated smoothing function, we do not have an explicit estimate of the detection time [3], and are unable to derive any confidence intervals (or bands) directly based on any known asymptotic theorem. Instead, we propose to use the Bootstrap method for this task[6]. Basically, we randomly select a sample of *N* viruses with replacement as a replicate. On the replicate sample, we fit the above GAM [2] to the sample, and estimate the detection time [3]. Repeating this bootstrapping procedure for, say 1000 times, we obtain a series of estimated detection times in all bootstrap samples, and use quantiles at 2.5% and 97.5% as estimated lower and upper confidence interval values at 95% significance level. A larger bootstrap sample size, e.g., 10,000, can improve the precision of estimating detection time, but requires much intensive computations, while the improvement in estimating detection time in fractions of a day is not practically essential given our interest in the resolution of "detection days".

6. Supervising learning to organize Omicron caseload percentages (OCP)

In exploration of temporal expansions of Omicron variants in each African country or one of the states/territories in the US, we obtained OCP values over time. As expected, Omicron variant has variable patterns of expanding in every population. To gain an insight into the overall pattern, we apply the unsupervised learning technique to daily estimates of the OCP values[7]. Computationally, this technique computes pairwise Eulidean distances of OCP between countries/regions. Using the "ward.D2" agglomeration method) [7], it will cluster those OCP patterns closer together, while keeping different patterns apart. Using the heatmap, it will display clustered OCP patterns for easy and intuitive

© 2022 Zhao LP et al. *JAMA Network Open.*

visualization. Note that alternative distances, such as “Manhattan” (absolute distance between two vectors) or “minkowski” (the p norm distance), have been experimented in the current applications, and resulting clusters are found rather robust.

© 2022 Zhao LP et al. *JAMA Network Open.*

eTable 1. Omicron Haplotype and Its Polymorphic Variations in the United States, October 1 to December 27, 2021

To assemble this set of Omicron haplotypes, we started with a base set of Omicron haplotypes with at least 14 mutations in Spike protein and at least three identical haplotypes observed. To allow for random mutations or missing residues, we included additional haplotypes if they are highly similar to one of base haplotypes. As a result, a total of 343 haplotypes were identified to form this set of Omicron haplotypes. We use S1-S28 to denote 28 PM (A67, T95, G339, S371, S373, S375, K417, N440, G446, S477, T478, E484, Q493, G496, Q498, N501, Y505, T547, D614, H655, N679, P681 , N764, D79, N856, Q954, N969, L981).

**ID**	**Spike**		1	2	3	4	5	6	7	8	9	10	11	12	13	14	15	16	17	18	19	20	21	22	23	24	25	26	27	28	mut
	**Freq**		A	T	G	S	S	S	K	N	G	S	T	E	Q	G	Q	N	Y	T	D	H	N	P	N	D	N	Q	N	L	#
h1	10278		V	I	D	L	P	F	N	K	S	N	K	A	R	S	R	Y	H	K	G	Y	K	H	K	Y	K	H	K	F	28
h2	449		V	I	D	L	P	F	.	K	S	N	K	A	R	S	R	Y	H	K	G	Y	K	H	K	Y	K	H	K	F	27
h3	411		V	I	D	L	P	F	.	.	.	N	K	A	R	S	R	Y	H	K	G	Y	K	H	K	Y	K	H	K	F	25
h4	56		V	I	D	L	P	F	N	K	S	N	K	A	R	S	R	Y	.	K	G	Y	K	H	K	Y	K	H	K	F	27
h5	47		V	I	D	L	P	F	.	K	.	N	K	A	R	S	R	Y	H	K	G	Y	K	H	K	Y	K	H	K	F	26
h6	46		V	I	D	L	P	F	N	K	S	N	K	A	R	S	.	.	.	K	G	Y	K	H	K	Y	K	H	K	F	25
h7	43		V	I	D	L	P	F	N	K	S	N	K	A	R	S	R	Y	H	K	G	Y	.	R	K	Y	K	H	K	F	27
h8	41		V	I	D	L	P	F	.	.	.	N	K	A	R	S	R	Y	H	K	G	Y	K	H	.	Y	K	H	K	F	24
h9	36		V	I	D	L	P	F	N	K	S	N	K	A	R	S	R	Y	H	K	G	Y	K	H	.	Y	K	H	K	F	27
h10	34		V	I	D	L	P	F	N	K	.	N	K	A	R	S	R	Y	H	K	G	Y	K	H	K	Y	K	H	K	F	27
h11	34		V	I	D	L	P	F	N	.	S	N	K	A	R	S	R	Y	H	K	G	Y	K	H	K	Y	K	H	K	F	27
h12	29		V	I	D	L	P	F	N	.	.	N	K	A	R	S	R	Y	H	K	G	Y	K	H	K	Y	K	H	K	F	26
h13	18		V	I	.	L	P	F	N	K	S	N	K	A	R	S	R	Y	H	K	G	Y	K	H	K	Y	K	H	K	F	27
h14	12		V	I	D	L	P	F	N	K	S	N	K	A	.	.	.	.	.	K	G	Y	K	H	K	Y	K	H	K	F	23
h15	11		V	I	D	L	P	F	.	.	S	N	K	A	R	S	R	Y	H	K	G	Y	K	H	K	Y	K	H	K	F	26

© 2022 Zhao LP et al. *JAMA Network Open.*

h16	11		V	I	D	L	P	F	N	K	S	N	K	A	.	.	.	.	H	K	G	Y	K	H	K	Y	K	H	K	F	24
h17	11		V	I	D	L	P	F	N	K	S	N	K	A	R	S	R	Y	H	K	G	X	K	H	K	Y	K	H	K	F	27
h18	11		V	I	D	L	P	F	X	K	S	N	K	A	R	S	R	Y	H	K	G	Y	K	H	K	Y	K	H	K	F	27
h19	10		.	I	D	L	P	F	N	K	S	N	K	A	R	S	R	Y	H	K	G	Y	K	H	K	Y	K	H	K	F	27
h20	10		V	I	D	L	P	F	N	K	S	N	K	A	R	S	R	Y	H	.	G	Y	K	H	K	Y	K	H	K	F	27
h21	9		V	I	D	L	P	F	N	K	S	N	K	.	R	S	R	Y	H	K	G	Y	K	H	K	Y	K	H	K	F	27
h22	9		V	I	D	L	P	F	N	K	S	.	.	.	.	.	.	.	.	K	G	Y	K	H	K	Y	K	H	K	F	20
h23	9		V	I	D	L	P	F	X	K	S	N	K	A	R	S	R	Y	H	K	G	Y	.	R	K	X	K	H	K	F	25
h24	9		V	.	D	L	P	F	N	K	S	N	K	A	R	S	R	Y	.	K	G	Y	K	H	K	Y	K	H	K	F	26
h25	8		V	I	D	L	P	.	N	K	S	N	K	A	R	S	R	Y	H	K	G	Y	K	H	K	Y	K	H	K	F	27
h26	8		V	I	D	.	.	.	N	K	S	N	K	A	R	S	R	Y	H	K	G	Y	K	H	K	Y	K	H	K	F	25
h27	8		V	I	.	.	.	.	.	.	.	.	.	.	.	.	.	.	.	K	G	Y	K	H	K	Y	K	H	K	F	13
h28	8		V	I	.	.	.	.	N	K	S	N	K	A	R	S	R	Y	H	K	G	Y	K	H	K	Y	K	H	K	F	24
h29	8		V	.	D	L	P	F	N	K	S	N	K	A	R	S	R	Y	H	K	G	Y	K	H	K	Y	K	H	K	F	27
h30	7		V	I	D	L	P	F	N	K	S	N	K	A	R	S	R	Y	H	K	G	Y	K	H	K	Y	.	H	K	F	27
h31	7		V	I	D	L	P	F	N	K	S	N	K	A	R	S	R	Y	H	K	G	Y	K	H	.	.	.	.	.	.	22
h32	7		V	I	D	.	.	.	.	K	S	N	K	A	R	S	R	Y	H	K	G	Y	K	H	K	Y	K	H	K	F	24
h33	7		V	I	.	.	.	.	.	.	S	N	K	A	R	S	R	Y	H	K	G	Y	K	H	K	Y	K	H	K	F	22
h34	6		V	I	D	L	P	F	.	.	.	N	K	A	R	S	R	Y	H	K	G	Y	K	H	X	Y	K	H	K	F	24
h35	6		V	I	D	L	P	F	N	K	S	N	K	A	R	S	R	Y	H	K	G	Y	K	H	K	Y	K	H	K	.	27
h36	6		V	I	D	L	P	F	N	K	S	N	K	A	R	S	R	Y	H	K	G	Y	.	H	K	Y	K	H	K	F	27
h37	6		V	I	D	L	P	F	N	K	S	N	K	.	.	.	.	.	H	K	G	Y	K	H	K	Y	K	H	K	F	23
h38	6		V	I	D	L	P	F	N	K	S	N	K	.	.	.	.	.	.	K	G	Y	K	H	K	Y	K	H	K	F	22
h39	6		V	I	D	L	P	.	N	K	S	N	K	A	R	S	R	Y	.	K	G	Y	K	H	K	Y	K	H	K	F	26
h40	6		V	I	.	.	.	.	.	K	S	N	K	A	R	S	R	Y	H	K	G	Y	K	H	K	Y	K	H	K	F	23
h41	5		V	I	D	L	P	F	.	K	S	.	.	.	.	.	.	.	.	K	G	Y	K	H	K	Y	K	H	K	F	19
h42	5		V	I	D	L	P	F	.	K	S	.	.	.	R	S	R	Y	H	K	G	Y	K	H	K	Y	K	H	K	F	24
h43	5		V	I	D	L	P	F	N	K	S	N	K	A	R	S	R	Y	H	K	G	Y	K	H	K	.	K	H	K	F	27
h44	5		V	I	D	L	P	F	N	K	S	N	K	A	R	S	R	Y	H	K	G	Y	K	H	.	.	K	H	K	F	26
h45	5		V	I	D	L	P	F	N	K	S	.	.	A	R	S	R	Y	H	K	G	Y	K	H	K	Y	K	H	K	F	26
h46	4		V	I	D	L	P	F	N	K	S	N	K	A	R	S	R	.	H	K	G	Y	K	H	K	Y	K	H	K	F	27

© 2022 Zhao LP et al. *JAMA Network Open.*

h47	4		V	I	D	L	P	F	N	K	S	N	K	A	R	S	R	Y	H	K	G	Y	K	H	K	Y	K	H	.	F	27
h48	4		V	I	.	L	P	F	.	K	S	N	K	A	R	S	R	Y	H	K	G	Y	K	H	K	Y	K	H	K	F	26
h49	3		V	I	D	L	P	F	.	K	.	N	K	A	R	S	R	Y	H	K	G	Y	K	H	.	Y	K	H	K	F	25
h50	3		V	I	D	L	P	F	.	K	.	N	K	A	R	S	R	Y	H	.	G	Y	K	H	K	Y	K	H	K	F	25
h51	3		V	I	D	L	P	F	.	K	S	N	K	A	R	S	R	Y	H	K	G	Y	K	H	K	.	K	H	K	F	26
h52	3		V	I	D	L	P	F	.	K	S	N	K	A	R	S	R	Y	H	K	G	Y	.	R	K	Y	K	H	K	F	26
h53	3		V	I	D	L	P	F	.	.	.	N	K	A	R	S	R	Y	H	K	G	Y	.	R	.	Y	K	H	K	F	23
h54	3		V	I	D	L	P	F	.	.	.	.	K	A	R	S	R	Y	H	K	G	Y	K	H	K	Y	K	H	K	F	24
h55	3		V	I	D	L	P	F	.	.	.	.	K	A	R	S	R	Y	H	K	G	Y	K	H	.	Y	K	H	K	F	23
h56	3		V	I	D	L	P	F	N	K	S	N	K	A	R	S	.	.	H	K	G	Y	K	H	K	Y	K	H	K	F	26
h57	3		V	I	D	L	P	F	N	K	S	N	K	A	R	S	.	Y	H	K	G	Y	K	H	K	Y	K	H	K	F	27
h58	3		V	I	D	L	P	F	N	K	S	N	K	A	R	S	R	Y	H	K	.	Y	K	H	K	Y	K	H	K	F	27
h59	3		V	I	D	L	P	F	N	K	S	N	K	A	R	S	R	Y	H	K	G	Y	K	H	K	Y	K	.	K	F	27
h60	3		V	I	D	L	P	F	N	K	S	N	K	A	R	S	R	Y	H	K	G	Y	K	H	K	Y	K	.	.	.	25
h61	3		V	I	D	L	P	F	N	K	S	N	K	A	R	S	R	Y	H	K	G	Y	K	R	K	Y	K	H	K	F	28
h62	3		V	I	D	L	P	F	X	K	S	N	K	A	R	S	R	Y	X	K	G	Y	K	H	K	Y	K	H	K	F	26
h63	2		.	I	D	L	P	F	N	K	S	N	K	A	R	S	R	Y	H	K	G	Y	.	H	K	Y	K	H	K	F	26
h64	2		.	.	D	L	P	F	N	K	S	N	K	A	R	S	R	Y	H	K	G	Y	K	H	K	Y	K	H	K	F	26
h65	2		V	I	D	L	P	F	.	K	S	N	K	A	.	.	.	.	.	K	G	Y	K	H	K	Y	K	H	K	F	22
h66	2		V	I	D	L	P	F	.	K	S	N	K	A	R	S	R	Y	H	K	G	Y	K	H	K	Y	K	H	K	.	26
h67	2		V	I	D	L	P	F	.	K	S	N	K	A	R	S	R	Y	H	.	G	Y	K	H	K	Y	K	H	K	F	26
h68	2		V	I	D	L	P	F	.	K	S	N	K	A	R	S	R	Y	.	K	G	Y	K	H	K	Y	K	H	K	F	26
h69	2		V	I	D	L	P	F	.	K	S	N	K	.	.	.	.	.	.	K	G	Y	K	H	K	Y	K	H	K	F	21
h70	2		V	I	D	L	P	F	.	K	S	N	K	.	R	S	R	Y	H	K	G	Y	K	H	K	Y	K	H	K	F	26
h71	2		V	I	D	L	P	F	.	.	.	.	K	A	R	S	R	Y	H	K	G	Y	X	X	.	Y	K	H	K	F	21
h72	2		V	I	D	L	P	F	.	.	.	.	K	X	R	S	R	Y	H	K	G	Y	.	R	.	Y	K	H	K	F	21
h73	2		V	I	D	L	P	F	.	.	.	.	.	.	.	.	.	.	.	K	G	Y	K	H	K	Y	K	H	K	F	17
h74	2		V	I	D	L	P	F	.	.	.	.	.	.	R	S	R	Y	H	K	G	Y	K	H	K	Y	K	H	K	F	22
h75	2		V	I	D	L	P	F	.	.	.	X	K	X	R	S	R	Y	H	K	G	Y	K	X	.	Y	K	H	K	F	21
h76	2		V	I	D	L	P	F	.	.	.	X	.	.	.	.	.	.	.	.	G	Y	K	H	.	Y	K	H	K	F	15
h77	2		V	I	D	L	P	F	N	K	.	N	K	A	R	S	R	Y	H	.	G	Y	K	H	K	Y	K	H	K	F	26

© 2022 Zhao LP et al. *JAMA Network Open.*

h78	2		V	I	D	L	P	F	N	K	S	N	K	A	.	S	R	Y	H	K	G	Y	K	H	K	Y	K	H	K	F	27
h79	2		V	I	D	L	P	F	N	K	S	N	K	A	R	S	R	.	.	K	G	Y	K	H	K	Y	K	H	K	F	26
h80	2		V	I	D	L	P	F	N	K	S	N	K	A	R	S	R	Y	H	K	G	.	.	.	K	Y	K	H	K	F	25
h81	2		V	I	D	L	P	F	N	K	S	N	K	A	R	S	R	Y	H	K	G	Y	K	.	K	Y	K	H	K	F	27
h82	2		V	I	D	L	P	F	N	K	S	N	K	.	.	S	R	Y	H	K	G	Y	K	H	K	Y	K	H	K	F	26
h83	2		V	I	D	L	P	F	N	K	S	N	.	.	R	S	R	Y	H	K	G	Y	K	H	K	Y	K	H	K	F	26
h84	2		V	I	D	L	P	F	N	K	S	.	K	A	R	S	R	Y	H	K	G	Y	K	H	K	Y	K	H	K	F	27
h85	2		V	I	D	L	P	F	N	.	.	N	K	A	R	S	R	Y	H	K	G	Y	K	H	.	Y	K	H	K	F	25
h86	2		V	I	D	L	P	F	N	.	.	.	K	.	.	.	.	.	.	K	G	Y	K	H	K	Y	K	H	K	F	19
h87	2		V	I	D	L	P	F	X	K	S	N	K	A	R	S	R	Y	X	K	G	Y	.	R	K	X	K	H	K	F	24
h88	2		V	I	D	L	P	F	X	K	S	N	K	A	R	S	R	Y	X	K	G	Y	.	R	K	Y	K	H	K	F	25
h89	2		V	I	D	L	P	F	X	X	X	N	K	A	R	S	R	Y	H	K	G	Y	K	H	.	Y	K	H	K	F	24
h90	2		V	I	D	L	P	F	X	X	X	N	K	A	R	S	R	Y	H	K	G	Y	K	H	X	Y	K	H	K	F	24
h91	2		V	I	D	L	P	.	.	K	S	N	K	A	R	S	R	Y	H	K	G	Y	K	H	K	Y	K	H	K	F	26
h92	2		V	I	D	L	.	F	N	K	S	N	K	A	R	S	R	Y	H	K	G	Y	K	H	K	Y	K	H	K	F	27
h93	2		V	I	D	.	.	.	.	.	.	.	K	.	.	.	.	.	.	K	G	Y	K	H	K	Y	K	H	K	F	15
h94	2		V	I	D	X	X	X	X	K	S	N	K	A	R	S	R	Y	H	K	G	Y	K	H	K	Y	K	H	K	F	24
h95	2		V	I	.	.	.	.	.	.	.	.	K	.	.	.	.	.	.	K	G	Y	K	H	K	Y	K	H	K	F	14
h96	2		V	I	.	.	.	.	N	K	S	N	K	A	R	S	R	Y	H	K	G	Y	K	H	.	.	K	H	K	F	22
h97	2		V	I	X	L	P	F	X	K	S	N	K	A	R	S	R	Y	X	K	G	Y	.	R	K	X	K	H	K	F	23
h98	2		V	.	D	L	P	F	N	K	S	N	K	A	R	S	.	.	.	K	G	Y	K	H	K	Y	K	H	K	F	24
h99	2		V	.	D	L	P	.	N	K	S	N	K	A	R	S	R	Y	.	K	G	Y	K	H	K	Y	K	H	K	F	25
h100	1		.	I	D	L	P	F	.	.	.	.	K	.	R	S	R	Y	H	K	G	Y	.	R	.	Y	.	H	.	F	18
h101	1		.	I	D	L	P	F	N	K	S	N	K	A	R	.	R	Y	H	.	G	Y	K	H	K	Y	.	H	K	.	23
h102	1		.	I	D	L	P	F	N	K	S	N	K	A	R	S	R	Y	H	K	G	Y	K	H	K	Y	K	.	K	F	26
h103	1		.	I	D	L	P	F	N	K	S	N	K	A	R	S	R	Y	H	K	G	Y	K	H	.	.	.	.	.	.	21
h104	1		.	I	D	L	P	F	N	K	S	N	K	A	R	S	R	Y	H	.	G	Y	K	H	K	Y	K	.	K	.	24
h105	1		.	I	D	L	P	F	N	K	S	.	.	A	R	S	R	Y	H	K	G	Y	K	H	K	Y	K	.	.	F	23
h106	1		.	I	D	L	.	.	N	K	S	N	K	A	R	S	.	.	.	K	G	Y	.	H	K	Y	.	H	K	F	20
h107	1		.	I	D	.	.	.	.	K	S	N	K	A	R	S	R	Y	H	K	G	Y	K	H	K	Y	K	H	K	F	23
h108	1		.	I	D	.	.	.	.	K	S	N	K	.	R	S	.	.	.	.	G	Y	K	H	K	Y	.	H	K	.	16

© 2022 Zhao LP et al. *JAMA Network Open.*

h109	1		.	I	D	.	.	.	N	K	S	N	K	A	R	S	R	Y	H	K	G	Y	K	H	K	Y	K	.	K	F	23
h110	1		.	I	.	L	P	F	N	K	S	N	K	A	R	S	R	Y	H	K	G	Y	K	H	K	Y	K	H	K	F	26
h111	1		.	I	.	L	P	F	N	K	S	.	.	A	R	S	R	Y	H	K	G	Y	K	H	K	Y	.	H	.	F	22
h112	1		.	I	.	.	.	F	N	.	.	.	K	.	.	S	R	Y	H	K	G	Y	K	H	K	Y	K	H	K	F	19
h113	1		.	I	X	L	P	F	.	.	.	N	K	A	R	S	R	Y	H	K	G	Y	K	.	K	Y	K	H	K	F	22
h114	1		.	I	X	L	P	F	.	.	.	N	K	A	R	S	R	Y	H	K	G	Y	K	.	K	Y	.	H	K	F	21
h115	1		.	I	X	L	P	F	.	.	.	N	K	A	R	S	R	Y	H	K	G	Y	K	.	K	Y	.	H	.	F	20
h116	1		.	I	X	L	P	F	X	K	S	N	K	A	R	S	R	Y	H	K	G	Y	.	.	K	.	.	H	K	F	21
h117	1		.	I	X	L	P	F	X	K	S	N	K	A	R	S	R	Y	H	K	G	Y	.	.	K	Y	K	H	K	F	23
h118	1		.	I	X	L	P	F	X	X	X	N	K	X	X	X	X	X	X	K	G	Y	.	H	X	Y	K	H	K	F	15
h119	1		.	.	D	F	P	F	N	K	.	N	K	A	R	.	R	Y	H	.	G	Y	K	H	K	Y	.	H	K	.	21
h120	1		.	.	D	L	P	F	.	K	S	N	K	A	R	S	R	Y	H	.	G	Y	K	H	.	.	K	H	K	F	22
h121	1		.	.	D	L	P	F	.	.	.	.	.	.	R	S	R	Y	H	K	.	.	.	.	.	.	.	H	K	F	13
h122	1		.	.	D	L	P	F	N	K	S	.	.	A	R	S	R	Y	H	K	G	Y	K	H	K	.	K	H	.	F	22
h123	1		.	.	D	L	P	F	X	X	X	N	K	A	R	S	R	Y	H	K	G	Y	K	H	.	.	.	H	K	F	20
h124	1		.	.	.	L	P	F	.	.	.	.	.	A	.	.	.	.	.	K	G	Y	K	.	K	Y	K	H	K	F	14
h125	1		.	.	.	L	P	F	N	K	S	N	K	A	R	S	.	.	.	K	G	Y	K	H	K	Y	K	H	K	F	22
h126	1		.	.	.	.	.	.	.	K	S	N	K	A	.	.	.	.	.	K	G	Y	K	H	.	.	K	H	K	F	14
h127	1		.	.	.	.	.	.	.	.	.	.	K	A	.	.	.	.	.	K	G	Y	K	H	K	Y	K	H	K	F	13
h128	1		.	.	.	.	.	.	.	.	.	.	K	.	.	.	.	.	.	K	G	Y	K	H	K	Y	K	H	K	F	12
h129	1		.	.	.	.	.	.	.	.	.	.	K	.	.	.	.	.	.	K	G	Y	K	H	.	.	K	H	K	F	10
h130	1		.	.	.	.	.	.	.	.	.	.	K	.	R	S	R	Y	H	K	G	Y	.	R	.	.	.	.	.	.	10
h131	1		.	.	.	.	.	.	N	K	S	N	K	A	R	S	R	Y	H	K	.	.	K	H	K	Y	.	H	K	F	19
h132	1		.	.	X	L	P	F	X	.	.	N	K	A	R	S	R	Y	H	K	G	Y	K	.	X	.	.	H	K	F	18
h133	1		V	E	D	L	P	F	N	K	S	N	K	A	R	S	R	Y	.	K	G	Y	K	H	K	Y	K	H	K	F	27
h134	1		V	I	D	F	.	.	X	X	X	N	K	A	R	S	R	Y	H	K	G	Y	.	H	.	Y	K	H	K	F	21
h135	1		V	I	D	L	P	F	.	K	.	N	K	A	.	S	R	Y	H	K	G	Y	K	H	K	Y	K	H	K	F	25
h136	1		V	I	D	L	P	F	.	K	.	N	K	A	.	S	R	Y	H	K	G	Y	K	H	K	Y	K	.	K	F	24
h137	1		V	I	D	L	P	F	.	K	.	N	K	A	R	S	R	Y	H	K	G	.	K	H	K	Y	K	H	K	F	25
h138	1		V	I	D	L	P	F	.	K	.	N	K	A	R	S	R	Y	H	.	G	Y	K	H	.	Y	K	H	K	F	24
h139	1		V	I	D	L	P	F	.	K	.	N	K	.	R	.	.	Y	.	.	G	Y	K	H	.	Y	K	H	K	F	20

© 2022 Zhao LP et al. *JAMA Network Open.*

h140	1		V	I	D	L	P	F	.	K	.	.	K	A	R	.	.	Y	H	.	G	Y	K	H	K	Y	K	H	K	F	22
h141	1		V	I	D	L	P	F	.	K	.	.	.	.	.	.	.	.	H	.	G	Y	K	H	K	Y	K	H	K	F	18
h142	1		V	I	D	L	P	F	.	K	.	.	.	.	R	S	R	Y	H	K	G	Y	K	H	.	Y	K	H	K	F	22
h143	1		V	I	D	L	P	F	.	K	S	N	K	A	.	.	.	.	H	K	G	Y	K	H	K	Y	K	H	K	F	23
h144	1		V	I	D	L	P	F	.	K	S	N	K	A	.	.	.	Y	H	K	G	Y	K	H	K	Y	K	H	K	F	24
h145	1		V	I	D	L	P	F	.	K	S	N	K	A	.	.	R	Y	H	K	G	Y	K	H	K	Y	K	H	K	F	25
h146	1		V	I	D	L	P	F	.	K	S	N	K	A	R	S	R	Y	H	K	G	Y	K	H	K	.	.	H	K	F	25
h147	1		V	I	D	L	P	F	.	K	S	N	K	A	R	S	R	Y	H	K	G	Y	.	H	K	Y	K	H	K	F	26
h148	1		V	I	D	L	P	F	.	K	S	N	K	A	R	S	R	Y	H	K	G	Y	.	R	.	.	K	H	K	F	24
h149	1		V	I	D	L	P	F	.	K	S	N	K	.	.	.	.	.	H	K	G	Y	K	H	K	Y	K	H	K	F	22
h150	1		V	I	D	L	P	F	.	K	S	N	K	.	.	S	R	Y	H	K	G	Y	K	H	K	Y	K	H	K	F	25
h151	1		V	I	D	L	P	F	.	K	S	N	K	.	R	.	R	.	.	K	G	Y	K	H	K	Y	K	H	K	F	23
h152	1		V	I	D	L	P	F	.	K	S	N	.	.	.	.	.	Y	H	K	G	Y	K	H	K	Y	K	H	K	F	22
h153	1		V	I	D	L	P	F	.	K	S	.	.	.	R	.	.	Y	H	K	G	Y	K	H	K	Y	K	H	K	F	22
h154	1		V	I	D	L	P	F	.	.	.	N	K	A	R	S	R	Y	H	K	G	Y	K	H	K	Y	K	X	K	F	24
h155	1		V	I	D	L	P	F	.	.	.	N	K	A	R	S	R	Y	H	K	G	Y	K	H	.	N	K	H	K	F	24
h156	1		V	I	D	L	P	F	.	.	.	N	K	A	R	S	R	Y	H	K	G	Y	K	H	.	X	X	H	K	F	22
h157	1		V	I	D	L	P	F	.	.	.	N	K	A	R	S	R	Y	H	K	G	Y	K	H	X	.	.	H	K	F	22
h158	1		V	I	D	L	P	F	.	.	.	N	K	A	R	S	R	Y	H	K	G	Y	K	X	.	Y	K	H	K	F	23
h159	1		V	I	D	L	P	F	.	.	.	N	K	A	R	S	R	Y	H	K	G	Y	.	H	.	Y	K	H	K	F	23
h160	1		V	I	D	L	P	F	.	.	.	N	K	A	R	S	R	Y	H	K	G	Y	.	H	.	Y	.	H	K	F	22
h161	1		V	I	D	L	P	F	.	.	.	N	K	A	R	S	R	Y	H	K	G	Y	X	X	.	Y	K	H	K	F	22
h162	1		V	I	D	L	P	F	.	.	.	N	K	A	R	S	R	Y	H	.	G	Y	K	H	.	Y	K	H	K	F	23
h163	1		V	I	D	L	P	F	.	.	.	N	K	A	R	S	R	Y	.	K	G	Y	K	H	K	Y	K	H	K	F	24
h164	1		V	I	D	L	P	F	.	.	.	N	K	.	.	S	R	Y	H	K	G	Y	K	H	.	Y	K	H	K	F	22
h165	1		V	I	D	L	P	F	.	.	.	.	K	.	.	.	.	.	.	K	G	Y	K	H	K	Y	K	H	K	F	18
h166	1		V	I	D	L	P	F	.	.	.	.	K	.	.	.	.	.	.	.	G	.	.	.	.	.	.	H	.	F	10
h167	1		V	I	D	L	P	F	.	.	.	.	K	.	.	.	.	.	.	.	G	Y	K	H	.	Y	K	H	K	F	16
h168	1		V	I	D	L	P	F	.	.	.	.	K	.	.	.	.	.	.	.	G	Y	K	R	K	Y	K	H	K	F	17
h169	1		V	I	D	L	P	F	.	.	.	.	K	X	R	S	R	Y	H	K	G	X	.	R	.	Y	K	H	K	F	20
h170	1		V	I	D	L	P	F	.	.	.	.	K	X	R	S	R	Y	H	K	G	Y	X	X	.	Y	K	H	K	F	20

© 2022 Zhao LP et al. *JAMA Network Open.*

h171	1		V	I	D	L	P	F	.	.	.	.	.	A	R	S	.	Y	H	K	G	Y	.	H	K	.	K	H	K	F	20
h172	1		V	I	D	L	P	F	.	.	.	.	.	A	R	S	R	Y	H	K	G	Y	K	H	K	Y	K	H	K	F	23
h173	1		V	I	D	L	P	F	.	.	.	.	.	.	.	.	.	.	.	K	.	.	.	.	.	.	.	H	K	F	10
h174	1		V	I	D	L	P	F	.	.	.	.	.	.	.	.	.	.	.	.	G	Y	K	H	.	Y	K	H	K	F	15
h175	1		V	I	D	L	P	F	.	.	.	.	.	.	.	.	.	X	.	.	G	Y	K	H	.	Y	K	H	K	F	15
h176	1		V	I	D	L	P	F	.	.	.	.	.	.	.	.	R	.	.	K	G	Y	K	H	K	.	.	H	K	F	16
h177	1		V	I	D	L	P	F	.	.	.	.	.	.	.	S	R	Y	H	.	G	Y	K	H	.	Y	K	.	K	F	18
h178	1		V	I	D	L	P	F	.	.	.	X	K	A	R	S	R	Y	H	K	G	Y	K	H	X	.	.	H	.	.	19
h179	1		V	I	D	L	P	F	.	.	.	X	K	A	R	S	R	Y	H	K	G	Y	K	X	.	Y	K	H	K	F	22
h180	1		V	I	D	L	P	F	.	.	.	X	K	X	R	S	R	Y	H	K	G	Y	K	H	K	Y	K	H	K	F	23
h181	1		V	I	D	L	P	F	.	.	.	X	K	X	R	S	R	Y	H	K	G	Y	K	X	X	Y	K	H	K	F	21
h182	1		V	I	D	L	P	F	.	X	X	X	X	A	R	S	R	Y	H	K	G	Y	K	H	X	Y	K	X	K	F	21
h183	1		V	I	D	L	P	F	N	K	.	N	K	A	R	S	R	Y	H	K	G	Y	K	H	.	Y	K	H	K	F	26
h184	1		V	I	D	L	P	F	N	K	.	N	K	.	.	.	.	.	H	K	G	Y	K	H	K	Y	K	H	K	F	22
h185	1		V	I	D	L	P	F	N	K	.	N	K	.	.	S	.	.	H	.	G	Y	K	H	.	Y	K	H	K	F	21
h186	1		V	I	D	L	P	F	N	K	.	N	.	A	.	S	R	Y	H	K	G	Y	K	H	K	Y	K	H	K	F	25
h187	1		V	I	D	L	P	F	N	K	.	.	K	.	R	S	R	Y	H	K	G	Y	K	H	K	Y	K	H	K	F	25
h188	1		V	I	D	L	P	F	N	K	S	N	K	A	.	.	.	.	.	K	G	Y	K	H	K	Y	K	H	K	.	22
h189	1		V	I	D	L	P	F	N	K	S	N	K	A	.	.	R	Y	H	K	G	Y	K	H	K	Y	K	H	K	F	26
h190	1		V	I	D	L	P	F	N	K	S	N	K	A	R	.	.	.	.	K	G	Y	K	H	K	Y	K	H	K	F	24
h191	1		V	I	D	L	P	F	N	K	S	N	K	A	R	.	.	Y	H	K	G	Y	K	H	K	Y	K	H	K	F	26
h192	1		V	I	D	L	P	F	N	K	S	N	K	A	R	.	R	Y	H	K	G	Y	K	H	K	Y	K	H	K	F	27
h193	1		V	I	D	L	P	F	N	K	S	N	K	A	R	S	.	.	.	K	G	Y	K	H	K	Y	K	H	.	F	24
h194	1		V	I	D	L	P	F	N	K	S	N	K	A	R	S	.	Y	.	K	G	Y	K	H	K	Y	K	H	K	F	26
h195	1		V	I	D	L	P	F	N	K	S	N	K	A	R	S	R	Y	H	K	.	.	K	H	K	Y	.	H	K	F	25
h196	1		V	I	D	L	P	F	N	K	S	N	K	A	R	S	R	Y	H	K	G	.	K	H	K	Y	K	H	K	F	27
h197	1		V	I	D	L	P	F	N	K	S	N	K	A	R	S	R	Y	H	K	G	.	.	.	.	.	K	H	K	F	23
h198	1		V	I	D	L	P	F	N	K	S	N	K	A	R	S	R	Y	H	K	G	.	.	R	K	Y	K	H	K	F	26
h199	1		V	I	D	L	P	F	N	K	S	N	K	A	R	S	R	Y	H	K	G	.	.	R	.	.	.	H	K	F	23
h200	1		V	I	D	L	P	F	N	K	S	N	K	A	R	S	R	Y	H	K	G	X	X	X	X	.	K	H	K	F	23
h201	1		V	I	D	L	P	F	N	K	S	N	K	A	R	S	R	Y	H	K	G	Y	K	H	K	.	.	H	.	.	24

© 2022 Zhao LP et al. *JAMA Network Open.*

h202	1		V	I	D	L	P	F	N	K	S	N	K	A	R	S	R	Y	H	K	G	Y	K	H	K	Y	K	H	.	.	26
h203	1		V	I	D	L	P	F	N	K	S	N	K	A	R	S	R	Y	H	K	G	Y	K	H	K	Y	K	H	X	X	26
h204	1		V	I	D	L	P	F	N	K	S	N	K	A	R	S	R	Y	H	K	G	Y	K	H	K	Y	K	.	.	F	26
h205	1		V	I	D	L	P	F	N	K	S	N	K	A	R	S	R	Y	H	K	G	Y	K	H	K	Y	X	H	K	F	27
h206	1		V	I	D	L	P	F	N	K	S	N	K	A	R	S	R	Y	H	K	G	Y	K	H	X	X	X	H	K	F	25
h207	1		V	I	D	L	P	F	N	K	S	N	K	A	R	S	R	Y	H	K	G	Y	K	R	K	.	.	H	.	F	25
h208	1		V	I	D	L	P	F	N	K	S	N	K	A	R	S	R	Y	H	K	G	Y	.	R	.	.	X	H	K	F	24
h209	1		V	I	D	L	P	F	N	K	S	N	K	A	R	S	R	Y	H	K	X	.	X	X	.	.	K	H	K	F	22
h210	1		V	I	D	L	P	F	N	K	S	N	K	A	R	S	R	Y	H	X	.	Y	K	H	K	Y	.	H	K	F	25
h211	1		V	I	D	L	P	F	N	K	S	N	K	A	R	S	R	Y	H	X	G	Y	K	.	K	Y	K	H	K	F	26
h212	1		V	I	D	L	P	F	N	K	S	N	K	.	.	.	.	Y	H	K	G	Y	K	H	K	Y	K	H	K	F	24
h213	1		V	I	D	L	P	F	N	K	S	N	K	.	.	.	.	Y	H	K	G	Y	.	.	K	Y	K	H	K	F	22
h214	1		V	I	D	L	P	F	N	K	S	N	K	.	R	S	.	.	.	K	G	Y	K	H	K	Y	K	H	K	F	24
h215	1		V	I	D	L	P	F	N	K	S	N	K	.	R	S	R	Y	.	K	G	Y	K	H	K	Y	K	H	K	F	26
h216	1		V	I	D	L	P	F	N	K	S	N	K	S	R	S	R	Y	H	K	G	Y	K	H	K	Y	K	H	K	F	28
h217	1		V	I	D	L	P	F	N	K	S	N	K	V	R	S	R	Y	H	K	G	Y	K	H	K	Y	K	H	K	F	28
h218	1		V	I	D	L	P	F	N	K	S	N	.	A	.	.	R	Y	H	K	G	Y	K	H	K	Y	K	H	K	F	25
h219	1		V	I	D	L	P	F	N	K	S	.	K	A	.	.	.	.	H	K	G	Y	K	H	K	Y	K	H	K	F	23
h220	1		V	I	D	L	P	F	N	K	S	.	K	.	.	.	.	.	.	K	G	Y	K	H	K	Y	K	H	K	F	21
h221	1		V	I	D	L	P	F	N	K	S	.	K	.	R	S	R	Y	.	K	G	Y	K	H	K	Y	K	H	K	F	25
h222	1		V	I	D	L	P	F	N	K	S	.	.	.	.	.	.	.	H	K	G	Y	K	H	K	Y	K	H	K	F	21
h223	1		V	I	D	L	P	F	N	K	S	.	.	.	.	.	R	Y	H	K	G	Y	K	H	K	Y	K	H	K	F	23
h224	1		V	I	D	L	P	F	N	K	S	.	.	.	.	S	R	Y	H	K	G	Y	K	H	K	Y	K	H	K	F	24
h225	1		V	I	D	L	P	F	N	K	S	.	.	.	R	S	R	Y	H	K	G	Y	K	H	K	Y	K	H	K	F	25
h226	1		V	I	D	L	P	F	N	K	S	X	X	X	X	X	X	X	X	.	G	Y	K	H	K	Y	K	H	K	F	19
h227	1		V	I	D	L	P	F	N	K	X	N	K	A	R	S	R	Y	H	K	G	Y	K	H	K	Y	K	H	K	F	27
h228	1		V	I	D	L	P	F	N	.	.	N	K	A	.	.	.	.	H	K	G	Y	K	H	K	Y	K	H	K	F	22
h229	1		V	I	D	L	P	F	N	.	.	N	K	A	R	S	R	Y	H	K	G	Y	.	R	.	Y	K	H	K	F	24
h230	1		V	I	D	L	P	F	N	.	.	N	K	.	R	S	R	Y	H	K	G	Y	K	H	K	Y	K	H	K	F	25
h231	1		V	I	D	L	P	F	N	.	.	.	K	.	.	.	.	.	.	.	G	Y	K	H	.	Y	K	H	K	F	17
h232	1		V	I	D	L	P	F	N	.	.	.	.	.	.	.	.	.	.	K	G	Y	K	H	K	Y	K	H	K	F	18

© 2022 Zhao LP et al. *JAMA Network Open.*

h233	1		V	I	D	L	P	F	N	.	S	N	K	A	R	.	.	Y	H	K	G	Y	K	H	K	Y	K	H	K	F	25
h234	1		V	I	D	L	P	F	N	.	S	N	K	A	R	S	R	Y	.	K	G	Y	K	H	K	Y	K	H	K	F	26
h235	1		V	I	D	L	P	F	N	.	S	.	K	.	.	.	.	.	.	K	G	Y	K	H	K	Y	K	H	K	F	20
h236	1		V	I	D	L	P	F	X	K	S	N	K	A	R	S	R	Y	H	K	G	X	X	X	K	.	X	H	K	F	22
h237	1		V	I	D	L	P	F	X	K	S	N	K	A	R	S	R	Y	H	K	G	Y	K	.	K	Y	K	H	K	F	26
h238	1		V	I	D	L	P	F	X	.	X	N	K	A	R	S	R	Y	H	K	G	Y	.	H	K	Y	K	H	K	F	24
h239	1		V	I	D	L	P	F	X	X	X	N	K	A	R	S	R	Y	H	K	G	.	.	R	.	.	.	.	.	.	17
h240	1		V	I	D	L	P	F	X	X	X	N	K	A	R	S	R	Y	H	K	G	X	X	X	X	X	K	H	K	F	20
h241	1		V	I	D	L	P	F	X	X	X	N	K	A	R	S	R	Y	H	K	G	Y	K	H	K	Y	K	H	K	F	25
h242	1		V	I	D	L	P	F	X	X	X	N	K	A	R	S	R	Y	H	K	G	Y	K	H	X	Y	K	H	X	X	22
h243	1		V	I	D	L	P	F	X	X	X	N	K	A	R	S	R	Y	H	K	G	Y	.	R	.	Y	K	H	K	F	23
h244	1		V	I	D	L	P	F	X	X	X	N	K	A	R	S	R	Y	H	K	G	Y	X	X	.	Y	K	H	K	F	22
h245	1		V	I	D	L	P	F	X	X	X	N	K	X	X	X	X	X	X	K	G	Y	K	H	X	X	K	H	K	F	17
h246	1		V	I	D	L	P	F	X	X	X	.	K	X	X	X	X	X	X	X	G	Y	K	H	K	Y	K	H	K	F	17
h247	1		V	I	D	L	P	F	X	X	X	X	X	A	R	S	R	Y	H	X	G	Y	K	H	X	X	K	H	X	F	19
h248	1		V	I	D	L	P	F	X	X	X	X	X	.	R	S	R	Y	H	K	G	Y	.	.	X	Y	K	H	K	F	19
h249	1		V	I	D	L	P	F	X	X	X	X	X	X	X	S	R	Y	H	K	G	Y	.	R	.	Y	K	H	K	F	19
h250	1		V	I	D	L	P	F	X	X	X	X	X	X	X	X	X	X	X	K	G	Y	.	.	.	Y	K	H	K	F	14
h251	1		V	I	D	L	P	.	N	K	S	N	K	A	R	S	*	Y	H	K	G	Y	K	H	K	Y	K	H	K	F	27
h252	1		V	I	D	L	.	.	.	.	.	.	K	A	R	S	.	Y	H	K	G	Y	.	H	K	Y	K	H	K	F	20
h253	1		V	I	D	L	.	.	.	.	.	.	K	A	R	S	R	Y	H	K	G	Y	.	H	.	Y	K	H	K	F	20
h254	1		V	I	D	P	P	F	N	K	S	N	K	A	R	S	.	.	H	K	G	Y	K	H	K	Y	K	H	K	F	26
h255	1		V	I	D	P	P	F	N	K	S	N	K	A	R	S	R	Y	H	K	G	Y	K	H	K	Y	K	H	K	F	28
h256	1		V	I	D	.	P	F	.	K	S	N	K	A	R	S	R	Y	H	K	G	Y	K	H	K	Y	K	H	K	F	26
h257	1		V	I	D	.	P	F	N	K	S	N	K	A	R	S	R	Y	H	K	G	Y	K	H	K	Y	K	H	K	F	27
h258	1		V	I	D	.	P	F	N	K	S	.	.	A	R	S	R	Y	H	K	G	Y	K	H	K	Y	K	H	K	F	25
h259	1		V	I	D	.	P	.	.	K	S	N	K	A	R	.	.	Y	H	K	G	Y	K	H	K	Y	K	H	K	F	23
h260	1		V	I	D	.	P	.	.	K	S	N	K	A	R	S	R	Y	H	K	G	Y	K	H	K	Y	K	H	K	F	25
h261	1		V	I	D	.	.	.	.	K	S	N	K	A	R	S	R	Y	.	K	G	Y	K	H	K	Y	K	H	K	F	23
h262	1		V	I	D	.	.	.	.	.	.	N	K	A	R	S	R	Y	H	K	G	Y	K	H	K	Y	K	H	K	F	22
h263	1		V	I	D	.	.	.	.	.	.	N	K	A	R	S	R	Y	.	K	G	Y	K	H	K	Y	K	H	K	F	21

© 2022 Zhao LP et al. *JAMA Network Open.*

h264	1		V	I	D	.	.	.	.	.	.	N	.	.	.	.	.	.	H	K	G	Y	K	H	K	Y	K	H	K	F	16
h265	1		V	I	D	.	.	.	.	.	.	.	K	.	R	S	R	Y	H	K	G	Y	K	H	.	Y	K	H	K	F	19
h266	1		V	I	D	.	.	.	.	.	.	.	.	.	.	.	.	.	.	K	G	Y	K	H	K	Y	K	H	K	F	14
h267	1		V	I	D	.	.	.	.	.	S	N	K	A	.	.	.	.	H	K	G	Y	K	H	K	Y	K	H	K	F	19
h268	1		V	I	D	.	.	.	.	.	S	N	K	A	R	.	.	.	.	K	G	Y	K	H	K	Y	K	H	K	F	19
h269	1		V	I	D	.	.	.	.	.	S	.	.	A	.	.	.	.	.	K	G	Y	K	H	K	Y	K	H	K	F	16
h270	1		V	I	D	.	.	.	N	K	S	N	K	A	R	S	R	Y	H	K	G	Y	.	H	K	Y	K	H	.	F	23
h271	1		V	I	D	.	.	.	N	K	S	.	.	A	R	S	.	.	.	K	G	Y	K	H	K	Y	K	H	.	F	19
h272	1		V	I	D	.	.	.	X	X	X	X	X	X	X	X	X	Y	H	K	G	Y	K	H	X	Y	K	H	K	X	14
h273	1		V	I	D	.	.	X	.	.	.	X	K	A	R	S	R	Y	H	K	G	Y	K	H	K	Y	K	H	K	F	21
h274	1		V	I	D	X	X	X	.	.	.	.	K	X	R	S	R	Y	H	K	G	Y	.	R	.	X	K	H	K	F	17
h275	1		V	I	D	X	X	X	.	.	.	.	K	X	R	S	R	Y	H	K	G	Y	X	X	.	Y	K	H	K	F	17
h276	1		V	I	D	X	X	X	.	.	.	X	K	A	R	S	R	Y	H	K	G	X	.	R	.	Y	X	X	K	F	16
h277	1		V	I	D	X	X	X	.	.	.	X	K	X	R	S	R	Y	H	K	G	Y	X	X	.	Y	K	H	K	F	17
h278	1		V	I	D	X	X	X	.	.	.	X	K	X	R	S	R	Y	H	K	G	Y	X	X	X	Y	K	H	K	F	17
h279	1		V	I	D	X	X	X	X	K	S	N	K	A	R	S	R	Y	H	K	G	Y	K	H	X	X	K	H	K	F	22
h280	1		V	I	D	X	X	X	X	K	S	N	K	A	R	S	R	Y	X	K	G	Y	K	H	K	X	K	H	K	F	22
h281	1		V	I	D	X	X	X	X	K	S	N	K	A	R	S	R	Y	X	K	G	Y	K	H	X	X	K	H	K	F	21
h282	1		V	I	D	X	X	X	X	K	S	N	K	A	R	S	R	Y	.	K	G	Y	K	H	K	X	X	H	K	F	21
h283	1		V	I	D	X	X	X	X	X	X	N	K	A	R	S	R	Y	H	K	G	Y	X	X	X	Y	X	H	K	F	18
h284	1		V	I	D	X	X	X	X	X	X	X	X	X	X	X	X	X	X	K	G	Y	K	H	K	Y	K	H	K	F	14
h285	1		V	I	.	L	P	F	.	K	.	N	K	A	R	.	R	Y	H	K	G	Y	K	H	K	Y	K	H	K	F	24
h286	1		V	I	.	L	P	F	N	K	.	N	K	A	R	S	R	Y	H	K	G	Y	K	H	K	Y	K	H	K	F	26
h287	1		V	I	.	L	P	F	N	K	S	N	K	A	.	S	R	Y	H	K	G	Y	K	H	K	Y	K	H	K	F	26
h288	1		V	I	.	L	P	F	N	K	S	N	K	A	R	S	.	Y	H	K	G	Y	K	H	K	Y	K	H	K	F	26
h289	1		V	I	.	L	P	F	N	K	S	N	K	A	R	S	R	Y	H	K	G	Y	.	H	K	Y	K	H	K	F	26
h290	1		V	I	.	L	P	F	N	K	S	N	K	A	R	S	R	Y	.	K	G	Y	K	H	K	Y	K	H	K	F	26
h291	1		V	I	.	L	P	F	N	K	S	N	K	.	.	.	.	.	.	K	G	Y	K	H	K	Y	K	H	K	F	21
h292	1		V	I	.	L	P	F	N	K	S	N	K	.	.	.	R	Y	H	K	G	Y	K	H	K	Y	K	H	K	F	24
h293	1		V	I	.	L	P	F	N	K	S	N	.	A	.	.	.	.	.	K	G	Y	K	H	K	Y	K	H	K	F	21
h294	1		V	I	.	L	P	F	N	.	S	N	K	A	.	S	R	Y	H	K	G	Y	K	H	K	Y	K	H	K	F	25

© 2022 Zhao LP et al. *JAMA Network Open.*

h295	1		V	I	.	L	P	.	N	K	S	N	K	A	R	S	R	Y	H	K	G	Y	K	H	K	Y	K	H	K	F	26
h296	1		V	I	.	L	.	.	.	.	.	N	K	.	.	.	.	.	.	.	G	Y	K	H	.	Y	K	H	K	F	14
h297	1		V	I	.	L	.	.	N	K	S	N	K	A	R	S	R	Y	H	K	G	Y	K	H	K	Y	K	H	K	F	25
h298	1		V	I	.	.	.	.	.	K	S	N	K	.	.	.	.	.	.	K	G	Y	K	H	K	Y	K	H	K	F	17
h299	1		V	I	.	.	.	.	.	.	.	N	K	A	R	S	R	Y	H	K	G	Y	K	H	K	Y	K	H	K	F	21
h300	1		V	I	.	.	.	.	.	.	.	N	K	.	.	.	.	.	.	K	G	Y	.	H	.	Y	K	H	K	F	13
h301	1		V	I	.	.	.	.	.	.	.	.	K	A	R	S	R	Y	H	K	G	Y	K	H	K	Y	K	H	K	F	20
h302	1		V	I	.	.	.	.	.	.	.	.	K	.	.	.	.	.	.	.	G	Y	K	H	.	Y	K	H	K	F	12
h303	1		V	I	.	.	.	.	.	.	.	.	.	.	.	.	.	.	H	K	G	Y	K	H	K	Y	K	H	K	F	14
h304	1		V	I	.	.	.	.	.	.	S	.	K	A	R	S	R	Y	H	.	G	Y	K	H	K	Y	K	H	K	F	20
h305	1		V	I	.	.	.	.	X	X	X	X	X	X	X	X	X	X	X	K	G	Y	K	H	X	.	K	H	K	F	11
h306	1		V	I	X	L	P	F	.	.	.	N	K	A	R	S	R	Y	H	K	G	Y	K	.	K	Y	.	H	K	F	22
h307	1		V	I	X	L	P	F	X	K	S	N	K	A	R	S	R	Y	H	K	G	.	.	R	X	X	K	H	K	F	22
h308	1		V	I	X	L	P	F	X	K	S	N	K	A	R	S	R	Y	H	K	G	Y	K	.	K	Y	K	H	K	F	25
h309	1		V	I	X	L	P	F	X	K	S	N	K	A	R	S	R	Y	X	K	G	.	.	R	X	X	K	H	K	F	21
h310	1		V	I	X	L	P	F	X	K	S	N	K	X	X	X	X	X	X	K	G	Y	.	R	X	X	K	H	K	F	17
h311	1		V	I	X	.	.	.	X	K	S	N	K	A	R	S	R	Y	H	K	G	Y	.	.	K	.	K	H	.	F	19
h312	1		V	I	X	X	X	X	.	.	.	N	K	A	R	S	R	Y	H	K	G	Y	K	H	K	Y	K	H	K	F	21
h313	1		V	I	X	X	X	X	N	K	S	N	K	A	R	S	R	Y	H	K	G	Y	X	R	.	.	K	H	K	F	21
h314	1		V	I	X	X	X	X	X	K	S	.	.	A	R	S	.	.	.	K	G	Y	K	H	K	Y	K	H	K	F	18
h315	1		V	I	X	X	X	X	X	K	S	.	.	A	R	S	R	Y	H	K	G	Y	K	H	K	Y	K	H	K	F	21
h316	1		V	I	X	X	X	X	X	X	X	N	K	A	R	S	R	Y	H	K	G	Y	K	H	X	Y	K	H	K	F	20
h317	1		V	I	X	X	X	X	X	X	X	X	X	X	X	.	.	X	X	K	G	Y	K	H	K	Y	K	H	K	F	13
h318	1		V	I	X	X	X	X	X	X	X	X	X	X	X	X	X	X	X	.	G	Y	K	H	X	Y	K	H	K	F	11
h319	1		V	.	D	L	P	F	.	K	S	N	K	A	R	S	R	Y	H	K	G	Y	K	H	K	Y	K	H	K	F	26
h320	1		V	.	D	L	P	F	N	K	S	N	K	A	R	S	.	.	.	K	G	Y	K	H	K	.	K	H	K	F	23
h321	1		V	.	D	L	P	F	N	K	S	N	K	.	.	.	R	Y	H	K	G	Y	.	H	K	Y	K	H	K	F	23
h322	1		V	.	D	L	P	F	N	K	S	N	K	.	.	S	R	Y	H	K	G	Y	K	H	K	Y	K	H	K	F	25
h323	1		V	.	D	L	P	F	N	K	S	.	.	A	R	S	R	Y	H	K	G	Y	K	H	K	Y	K	H	K	F	25
h324	1		V	.	D	L	P	.	N	K	S	N	K	A	R	S	R	Y	H	K	G	Y	K	H	K	Y	K	H	K	F	26
h325	1		V	.	D	L	P	.	N	K	S	.	K	A	.	S	R	Y	H	K	G	Y	K	H	K	Y	K	H	K	F	24

© 2022 Zhao LP et al. *JAMA Network Open.*

h326	1		V	.	.	.	.	.	.	.	.	.	K	A	.	.	.	.	.	K	G	Y	.	.	K	Y	K	H	K	F	12
h327	1		V	X	D	L	P	F	N	K	S	N	K	A	R	X	X	X	X	K	G	Y	K	H	K	Y	K	H	K	F	23
h328	1		X	I	D	L	P	F	.	.	.	N	K	A	R	S	R	Y	H	K	G	Y	K	H	K	Y	K	X	X	X	21
h329	1		X	I	D	L	P	F	.	.	.	N	K	A	R	S	R	Y	H	K	G	Y	K	H	.	Y	K	H	K	F	23
h330	1		X	I	D	L	P	F	.	.	.	N	K	A	R	S	R	Y	H	K	G	Y	X	X	.	Y	K	H	K	F	21
h331	1		X	I	D	L	P	F	.	.	.	.	K	.	R	S	R	Y	H	K	G	Y	K	H	X	X	K	.	.	.	17
h332	1		X	I	D	L	P	F	X	X	X	N	K	A	R	S	R	X	H	X	G	Y	K	H	.	Y	K	X	X	X	18
h333	1		X	I	D	.	.	.	.	K	S	X	K	X	X	X	X	X	X	K	G	X	X	X	X	X	K	X	K	F	10
h334	1		X	I	D	X	X	X	.	.	.	.	K	X	R	S	R	Y	H	K	G	X	X	X	.	X	X	X	K	F	12
h335	1		X	I	D	X	X	X	.	.	.	.	K	X	R	S	R	Y	H	K	G	Y	.	R	.	Y	K	H	K	F	17
h336	1		X	I	D	X	X	X	.	.	.	.	K	X	R	S	R	Y	H	K	G	Y	X	X	.	Y	K	H	.	.	14
h337	1		X	I	D	X	X	X	.	.	.	X	K	X	R	S	R	Y	H	K	G	Y	X	X	.	Y	K	H	K	F	16
h338	1		X	X	D	L	P	F	.	.	.	X	K	X	X	X	R	Y	H	K	G	X	X	X	X	Y	K	X	K	F	14
h339	1		X	X	D	L	P	F	N	K	S	N	K	A	R	S	R	Y	H	K	G	Y	K	H	.	.	.	.	.	.	20
h340	1		X	X	D	L	P	F	N	K	S	N	K	A	R	X	R	Y	H	K	G	Y	K	H	.	.	.	.	.	.	19
h341	1		X	X	D	L	P	F	X	X	X	X	K	X	X	X	X	X	X	X	G	X	X	X	.	Y	K	H	K	F	11
h342	1		X	X	D	L	P	F	X	X	X	X	X	X	X	X	X	X	X	X	G	Y	K	H	X	Y	X	H	K	F	12
h343	1		X	X	X	L	P	F	X	X	X	X	X	X	X	X	X	X	X	X	G	Y	.	.	X	Y	K	H	K	F	10

© 2022 Zhao LP et al. *JAMA Network Open.*

eTable 2. Distribution of Lineages Among Omicron Cases in Africa and the United States

Lineage	Africa	US
BA.1	2578	11900
BA.2	19	1
BA.3	13	2
B.1	1	4
B.1.1	5	2
B.1.1.189	7	
B.1.1.409	3	
B.1.1.528	1	
B.1.1.529	25	44
B.1.576	1	
B.1.606	1	
B.1.617.2	2	
B.1.1.161		1
B.1.1.523		1
B.6	2	
AY.1	1	1
AY.103		1
AY.113	1	
AY.42		1
UNK	38	183

© 2022 Zhao LP et al. *JAMA Network Open.*

eTable 3. Frequency Distributions of Gender, Collection Date, and Collection Countries Among non-Omicron Cases and Omicron Cases in This Study, With Positive Percentage Computed, Among a Total of 63 686 COVID-19 Cases From African Countries

**Variable**	**Negative**	**Positive**		**Pos %**
N=	60988	2698		
**Gender**				
Female	27722 (45.45)	1426 (52.85)		4.89
Male	25917 (42.5)	1152 (42.7)		4.26
UNK	7349 (12.05)	120 (4.45)		1.61
**Collection Date**				
2020-01	3 (0.00)			
2020-02	13 (0.02)			
2020-03	378 (0.62)			
2020-04	801 (1.31)			
2020-05	1175 (1.93)			
2020-06	1315 (2.16)			
2020-07	2207 (3.62)			
2020-08	1087 (1.78)			
2020-09	1072 (1.76)			
2020-01	1420 (2.33)			
2020-11	1874 (3.07)			
2020-12	2968 (4.87)	1 (0.04)		0.03
2021-01	4936 (8.09)			
2021-02	3163 (5.19)			
2021-03	3773 (6.19)			
2021-04	2343 (3.84)			
2021-05	3200 (5.25)			
2021-06	6041 (9.91)			

© 2022 Zhao LP et al. *JAMA Network Open.*

2021-07	8192 (13.43)			
2021-08	7452 (12.22)			
2021-09	4138 (6.78)	1 (0.04)		0.02
2021-01	1885 (3.09)	3 (0.11)		0.16
2021-11	1293 (2.12)	1243 (46.07)		49.01
2021-12	259 (0.42)	1450 (53.74)		84.84
**Country in Africa**				
Angola	1054 (1.73)			
Benin	739 (1.21)			
Botswana	1781 (2.92)	388 (14.38)		17.89
Burkina.Faso	587 (0.96)			
Cameroon	551 (0.90)			
Democratic.Republic.of.the.Congo	629 (1.03)			
Egypt	1359 (2.23)	1 (0.04)		0.07
Ethiopia	524 (0.86)			
Gambia	922 (1.51)			
Ghana	2264 (3.71)	77 (2.85)		3.29
Kenya	5465 (8.96)	35 (1.3)		0.64
Madagascar	789 (1.29)			
Malawi	773 (1.27)	34 (1.26)		4.21
Mayotte	805 (1.32)			
Morocco	458 (0.75)	21 (0.78)		4.38
Mozambique	910 (1.49)	17 (0.63)		1.83
Nigeria	3626 (5.95)	84 (3.11)		2.26
Reunion	5096 (8.36)	7 (0.26)		0.14
Rwanda	670 (1.1)			
Senegal	704 (1.15)	29 (1.07)		3.96
Seychelles	538 (0.88)			

© 2022 Zhao LP et al. *JAMA Network Open.*

South.Africa	24132 (39.57)	1908 (70.72)		7.33
Uganda	926 (1.52)	18 (0.67)		1.91
Zambia	876 (1.44)	46 (1.7)		4.99
Zimbabwe	705 (1.16)			
Countries (<500 Covid Cases)	4105 (6.73)	33 (1.22)		0.80

© 2022 Zhao LP et al. *JAMA Network Open.*

eTable 4. Frequency Distributions of Gender, Collection Date, and Collection Countries Among non-Omicron Cases and Omicron Cases in This Study, With Positive Percentage Computed, Among a Total of 531 827 COVID-19 Cases From the United States

Variable	Neg	Pos		Pos %
N	519,686	12,141		
**Gender**				
Female	165490 (31.84)	4747 (39.1)		2.79
Male	157210 (30.25)	4098 (33.75)		2.54
UNK	196986 (37.9)	3296 (27.15)		1.65
**Collection Date**				
2021-10	218850 (42.11)			
2021-11	228386 (43.95)	119 (0.98)		0.05
2021-12	72450 (13.94)	12022 (99.02)		14.23
**Collection State**				
Alabama	930 (0.18)	15 (0.12)		1.59
Alaska	1944 (0.37)	4 (0.03)		0.21
Arizona	15452 (2.97)	168 (1.38)		1.08
Arkansas	1669 (0.32)	3 (0.02)		0.18
California	78404 (15.09)	3426 (28.22)		4.19
Colorado	49426 (9.51)	396 (3.26)		0.79
Connecticut	8596 (1.65)	162 (1.33)		1.85
Delaware	2001 (0.39)	4 (0.03)		0.20
District of Columbia	1697 (0.33)	307 (2.53)		15.32

© 2022 Zhao LP et al. *JAMA Network Open.*

Florida	9690 (1.86)	275 (2.27)		2.76
Georgia	4687 (0.9)	274 (2.26)		5.52
Hawaii	1364 (0.26)	93 (0.77)		6.38
Idaho	3166 (0.61)	21 (0.17)		0.66
Illinois	13425 (2.58)	248 (2.04)		1.81
Indiana	11120 (2.14)	93 (0.77)		0.83
Iowa	4365 (0.84)	31 (0.26)		0.71
Kansas	4289 (0.83)	20 (0.16)		0.46
Kentucky	4168 (0.8)	38 (0.31)		0.90
Louisiana	1742 (0.34)	333 (2.74)		16.05
Maine	2837 (0.55)	9 (0.07)		0.32
Maryland	8982 (1.73)	395 (3.25)		4.21
Massachusetts	40270 (7.75)	288 (2.37)		0.71
Michigan	19568 (3.77)	21 (0.17)		0.11
Minnesota	34004 (6.54)	76 (0.63)		0.22
Mississippi	1442 (0.28)	13 (0.11)		0.89
Missouri	4823 (0.93)	7 (0.06)		0.14
Montana	2719 (0.52)	5 (0.04)		0.18
Nebraska	5447 (1.05)	21 (0.17)		0.38
Nevada	6691 (1.29)	23 (0.19)		0.34
New Hampshire	3297 (0.63)	3 (0.02)		0.09
New Jersey	11479 (2.21)	203 (1.67)		1.74
New Mexico	7300 (1.4)	3 (0.02)		0.04
New York	23536 (4.53)	1975 (16.27)		7.74
North Carolina	10833 (2.08)	145 (1.19)		1.32
North Dakota	2898 (0.56)	4 (0.03)		0.14
Ohio	9686 (1.86)	376 (3.1)		3.74
Oklahoma	814 (0.16)	4 (0.03)		0.49
Oregon	4935 (0.95)	83 (0.68)		1.65
Pennsylvania	13210 (2.54)	96 (0.79)		0.72

© 2022 Zhao LP et al. *JAMA Network Open.*

Rhode Island	3507 (0.67)	15 (0.12)		0.43
South Carolina	2732 (0.53)	56 (0.46)		2.01
South Dakota	907 (0.17)	1 (0.01)		0.11
Tennessee	4602 (0.89)	35 (0.29)		0.75
Texas	16336 (3.14)	1096 (9.03)		6.29
US Islands	236 (0.05)	8 (0.07)		3.28
Utah	9338 (1.8)	20 (0.16)		0.21
Vermont	7405 (1.42)	7 (0.06)		0.09
Virginia	5088 (0.98)	94 (0.77)		1.81
Washington	13643 (2.63)	688 (5.67)		4.80
West Virginia	6415 (1.23)	19 (0.16)		0.30
Wisconsin	13477 (2.59)	435 (3.58)		3.13
Wyoming	3094 (0.6)	6 (0.05)		0.19

© 2022 Zhao LP et al. *JAMA Network Open.*

eTable 5. Frequencies of Observed Omicron viruses in African Countries: Botswana, Egypt, Ghana, Kenya, Malawi, Morocco, Mozambique, Nigeria, Reunion, Senegal, South Africa, Uganda, Zambia and Other Countries From October 31, 2020, to December 28, 2021

Other countries coded as c1, c2, ..., c13 and O, respectively.

**Date**	c1	c2	c3	c4	c5	c6	c7	c8	c9	c10	c11	c12	c13	O		Total
2020-12-31											1					**1**
Nine months																
2021-09-30											1					**1**
2021-10-12											1					**1**
2021-10-17								1								**1**
2021-10-24											1					**1**
2021-11-02								1			1					**2**
2021-11-05											1					**1**
2021-11-08											1					**1**
2021-11-09										1	3					**4**
2021-11-10			2								5					**7**
2021-11-11	5										4					**9**
2021-11-12											4					**4**
2021-11-13											2					**2**
2021-11-14											4					**4**
2021-11-15	1										18					**19**
2021-11-16											54					**54**
2021-11-17	1										56					**57**
2021-11-18											36					**36**
2021-11-19											38					**38**
2021-11-20								2			28					**30**
2021-11-21	3		1								15					**19**

© 2022 Zhao LP et al. *JAMA Network Open.*

2021-11-22	2		5					1	1		72					**81**
2021-11-23	11		2					1			75			5		**94**
2021-11-24	1		12					2			116			3		**134**
2021-11-25	4		18							1	91					**114**
2021-11-26	6		7	3			2	1			74			1		**94**
2021-11-27	1		2	1			1	1			22			1		**29**
2021-11-28	2		2					2			23					**29**
2021-11-29	4		1				3	4	1		159	7				**179**
2021-11-30	7		4				11	1		1	173	1	3	1		**202**
2021-12-01	29		1	4				4	1		177		1			**217**
2021-12-02	12			2	1			3			147	1	4			**170**
2021-12-03	3			2	4			1			122					**132**
2021-12-04	4			7				3		1	39		1			**55**
2021-12-05	2			1	2			3			16		2			**26**
2021-12-06	7		1	11	4			7			80		3			**113**
2021-12-07	38		4	3	1			12	4	3	43		5			**113**
2021-12-08	28		4		1			3		1	20	4	3			**64**
2021-12-09	15	1	5	1				1		4	44		7			**78**
2021-12-10	9		2					11		2	50		4	1		**79**
2021-12-11	15		3					6		1	21		6			**52**
2021-12-12	38							3		1	5					**47**
2021-12-13	99				3			5		1	6		6			**120**
2021-12-14	8				3	1		4		1	4	5	1	1		**28**
2021-12-15	9				3	2				1	15					**30**
2021-12-16	16		1		9	2		1			3					**32**
2021-12-17					3					2	16			2		**23**
2021-12-18	7									5	2			1		**15**
2021-12-19	1									3	7					**11**
2021-12-20						4					12			2		**18**
2021-12-21						3								4		**7**
2021-12-22						8								3		**11**

© 2022 Zhao LP et al. *JAMA Network Open.*

2021-12-24						1										**1**
2021-12-27														2		**2**
2021-12-28														6		**6**
Total	388	1	77	35	34	21	17	84	7	29	1908	18	46	33		**2698**

© 2022 Zhao LP et al. *JAMA Network Open.*

eTable 6. First 3 Omicron Cases From Eastern Cape, South Africa

The dot "." at a position represents the same amino acid in the fully-fledged Omicron haplotype (in red). The missing amino acid is marked "X" (blue)

Date		1	2	3	4	5	6	7	8	9	10	11	12	13	14	15	16	17	18	19	20	21	22	23	24	25	26	27	28
2020-12-31		V	I	.	.	.	.	N	x	.	.	.	K	.	.	.	Y	.	x	G	Y	K	H	x	.	x	H	K	F
2021-10-12		V	I	D	L	P	F	N	K	S	.	K	.	.	.	.	Y	.	K	G	Y	K	H	K	Y	K	H	K	F
2021-10-24		V	I	D	L	P	F	N	K	S	N	K	A	R	S	R	Y	H	K	G	Y	K	H	K	Y	K	H	K	F

© 2022 Zhao LP et al. *JAMA Network Open.*

eTable 7. Omicron Haplotype and Its Polymorphic Variations in African Countries, January 1, 2020, to December 28, 2021

We use S1-S28 to denote 28 PM (A67, T95, G339, S371, S373, S375, K417, N440, G446, S477, T478, E484, Q493, G496, Q498, N501, Y505, T547, D614, H655, N679, P681, N764, D79, N856, Q954, N969, L981).

**ID**	**Freq**		1	2	3	4	5	6	7	8	9	10	11	12	13	14	15	16	17	18	19	20	21	22	23	24	25	26	27	28	mut
	REF		A	T	G	S	S	S	K	N	G	S	T	E	Q	G	Q	N	Y	T	D	H	N	P	N	D	N	Q	N	L	#
h1	2393		V	I	D	L	P	F	N	K	S	N	K	A	R	S	R	Y	H	K	G	Y	K	H	K	Y	K	H	K	F	28
h2	26		V	I	D	L	P	F	.	K	S	N	K	A	R	S	R	Y	H	K	G	Y	K	H	K	Y	K	H	K	F	27
h3	24		V	I	D	.	.	.	.	K	S	N	K	A	R	S	R	Y	H	K	G	Y	K	H	K	Y	K	H	K	F	24
h4	15		.	.	D	F	P	F	N	K	.	N	K	A	R	.	R	Y	H	.	G	Y	K	H	K	Y	.	H	K	.	21
h5	14		V	I	D	F	P	F	N	K	S	N	K	A	R	.	R	Y	H	.	G	Y	K	H	K	Y	.	H	K	.	24
h6	13		V	I	.	.	.	.	.	K	S	N	K	A	R	S	R	Y	H	K	G	Y	K	H	K	Y	K	H	K	F	23
h7	11		V	I	D	L	.	.	N	K	S	.	.	.	R	S	R	Y	H	K	G	Y	.	.	K	Y	K	H	K	F	21
h8	6		V	I	D	L	P	F	N	K	S	N	K	A	R	S	R	Y	H	K	G	Y	K	H	.	.	.	.	.	.	22
h9	6		V	I	D	L	P	.	N	K	S	N	K	A	R	S	R	Y	.	K	G	Y	K	H	K	Y	K	H	K	F	26
h10	6		V	I	D	P	P	F	N	K	S	N	K	A	R	S	R	Y	H	K	G	Y	K	H	K	Y	K	H	K	F	28
h11	6		V	I	.	.	.	.	.	.	S	N	K	A	R	S	R	Y	H	K	G	Y	K	H	K	Y	K	H	K	F	22
h12	4		.	.	D	F	P	F	N	K	.	N	K	A	R	.	R	Y	.	.	G	Y	K	H	K	Y	.	H	K	.	20
h13	4		V	I	D	L	P	F	N	.	.	.	K	.	.	.	.	.	.	K	G	Y	K	H	K	Y	K	H	K	F	19
h14	4		V	I	D	L	.	.	.	.	.	.	.	.	R	S	R	Y	H	K	G	Y	.	.	K	Y	K	H	K	F	18
h15	4		V	I	.	X	X	X	X	X	X	X	X	X	X	X	X	X	X	K	G	Y	.	.	.	Y	K	H	K	F	10
h16	3		V	I	D	L	P	F	.	.	.	N	K	A	R	S	R	Y	H	K	G	Y	K	H	K	Y	K	H	K	F	25
h17	3		V	I	D	L	P	F	.	.	S	N	K	A	R	S	R	Y	H	K	G	Y	K	H	.	Y	K	H	K	F	25
h18	3		V	I	D	L	P	F	N	K	S	N	K	A	R	.	.	.	.	K	G	Y	K	H	K	Y	K	H	K	F	24
h19	3		V	I	D	L	P	F	N	K	S	N	K	A	R	S	R	Y	H	K	G	Y	K	H	K	Y	K	.	K	F	27
h20	3		V	I	D	L	P	F	N	K	S	N	K	A	R	S	R	Y	.	K	G	Y	K	H	K	Y	K	H	K	F	27
h21	3		V	I	D	L	P	.	.	K	S	N	K	A	R	S	R	Y	H	K	G	Y	K	H	K	Y	K	H	K	F	26
h22	3		V	I	D	.	.	.	N	K	S	N	K	A	R	S	R	Y	H	K	G	Y	K	H	K	Y	K	H	K	F	25

© 2022 Zhao LP et al. *JAMA Network Open.*

h23	2		.	.	D	F	P	F	N	K	.	N	.	A	R	.	R	Y	H	.	G	Y	K	H	K	Y	.	H	K	.	20
h24	2		.	.	D	L	P	F	N	K	S	N	K	A	R	S	R	Y	H	K	G	Y	K	H	.	.	.	.	.	.	20
h25	2		V	I	D	L	P	F	.	K	S	N	K	A	R	S	R	Y	H	K	G	Y	K	H	.	Y	K	H	K	F	26
h26	2		V	I	D	L	P	F	N	K	S	N	K	A	R	.	.	Y	H	K	G	Y	K	H	K	Y	K	H	K	F	26
h27	2		V	I	D	L	P	F	N	K	S	N	K	A	R	S	R	Y	H	K	G	Y	K	H	K	.	K	H	K	F	27
h28	2		V	I	D	L	P	F	N	K	S	N	K	A	R	S	R	Y	H	K	G	Y	K	H	K	.	.	.	.	.	23
h29	2		V	I	D	L	P	F	N	K	S	N	K	A	R	S	R	Y	H	K	G	Y	K	H	K	Y	K	H	.	F	27
h30	2		V	I	D	L	P	F	N	K	S	N	K	A	R	S	R	Y	H	K	G	Y	K	R	.	.	.	.	.	.	22
h31	2		V	I	D	L	P	F	N	K	S	N	K	A	R	S	R	Y	H	K	G	Y	.	R	.	.	.	.	.	.	21
h32	2		V	I	D	L	P	F	N	K	S	.	.	.	.	.	.	.	.	K	G	Y	K	H	K	Y	K	H	K	F	20
h33	2		V	I	D	L	P	F	N	.	.	N	K	A	R	S	R	Y	H	K	G	Y	K	H	K	Y	K	H	K	F	26
h34	2		V	I	D	L	P	F	N	.	S	N	K	A	R	S	R	Y	H	K	G	Y	K	H	K	Y	K	H	K	F	27
h35	2		V	I	D	L	P	F	N	.	S	.	.	.	.	.	.	.	.	K	G	Y	K	H	K	Y	K	H	K	F	19
h36	2		V	I	D	L	P	F	X	X	X	N	K	A	R	S	R	Y	H	K	G	Y	K	H	X	Y	K	H	K	F	24
h37	2		V	I	D	L	.	.	.	.	.	.	K	.	R	S	R	Y	H	K	G	Y	.	.	K	Y	K	H	K	F	19
h38	2		V	I	D	L	.	.	N	K	S	.	.	.	R	S	R	Y	H	K	G	Y	K	H	K	Y	K	H	K	F	23
h39	2		V	I	D	L	.	.	N	K	S	.	.	.	R	S	R	Y	H	K	G	Y	.	.	K	Y	K	H	.	.	19
h40	2		V	I	D	P	P	F	N	K	S	N	K	A	R	S	R	Y	H	K	G	Y	.	.	K	Y	K	H	K	F	26
h41	2		V	I	.	L	P	F	N	K	S	N	K	A	R	S	R	Y	H	K	G	Y	K	H	K	Y	K	H	K	F	27
h42	2		V	I	.	.	.	.	.	.	.	.	K	.	.	.	.	.	.	K	G	.	.	R	K	Y	X	X	K	F	10
h43	2		V	I	.	.	.	.	N	K	S	N	K	A	R	S	R	Y	H	K	G	Y	K	H	K	Y	K	H	K	F	24
h44	1		.	I	D	L	P	F	N	K	S	N	K	A	R	S	R	Y	H	K	G	Y	K	H	.	.	.	.	.	.	21
h45	1		.	I	D	L	P	F	N	K	S	N	K	A	R	S	R	Y	H	K	G	Y	.	H	.	.	.	.	.	.	20
h46	1		.	.	D	F	P	F	N	K	.	N	K	A	R	.	R	.	.	.	G	Y	K	H	K	Y	.	H	K	.	19
h47	1		.	.	D	L	P	F	N	K	S	N	K	A	R	S	.	Y	H	K	G	.	.	R	.	.	.	.	.	.	17
h48	1		.	.	D	L	P	F	N	K	S	N	K	A	R	S	R	Y	H	K	G	.	.	R	.	.	.	.	.	.	18
h49	1		.	.	D	L	P	F	N	.	S	N	K	A	R	S	.	Y	H	K	G	.	.	R	.	.	.	.	.	.	16
h50	1		.	.	D	L	P	F	N	.	S	N	K	A	R	S	R	.	H	K	G	.	.	H	.	.	.	.	.	.	16
h51	1		.	.	D	L	P	.	N	K	S	N	K	A	R	S	R	Y	H	K	G	.	.	H	.	.	.	.	.	.	17
h52	1		.	.	D	L	P	.	N	.	S	N	K	A	R	S	.	.	H	K	G	Y	.	H	.	.	.	.	.	.	15
h53	1		.	.	D	P	.	.	N	K	S	N	K	A	R	S	.	.	H	.	G	.	.	R	.	.	.	.	.	.	13

© 2022 Zhao LP et al. *JAMA Network Open.*

h54	1		.	.	D	P	.	.	N	.	S	N	K	.	.	S	.	.	.	K	G	.	.	R	.	.	.	.	.	.	10
h55	1		.	.	D	.	.	.	.	.	.	.	K	.	X	X	R	Y	H	K	G	Y	.	R	.	Y	K	H	K	F	14
h56	1		.	.	D	.	.	.	N	.	.	N	K	A	R	S	.	.	H	K	G	Y	.	R	.	.	.	.	.	.	12
h57	1		.	.	D	.	.	.	N	.	S	N	K	.	R	S	.	.	H	K	G	.	.	R	.	.	.	.	.	.	11
h58	1		V	I	D	F	P	F	N	K	.	N	K	A	R	.	R	Y	H	K	G	Y	K	H	K	Y	K	H	K	F	26
h59	1		V	I	D	F	P	F	N	K	S	N	K	A	R	S	R	Y	H	K	G	Y	K	H	K	Y	K	H	K	F	28
h60	1		V	I	D	L	P	F	.	K	.	N	K	A	R	S	R	Y	H	K	G	Y	K	H	K	Y	K	H	K	F	26
h61	1		V	I	D	L	P	F	.	K	S	N	K	A	R	S	R	Y	H	K	G	Y	K	H	K	Y	K	H	K	.	26
h62	1		V	I	D	L	P	F	.	K	S	N	K	A	R	S	R	Y	H	K	G	Y	.	.	.	Y	K	H	.	F	23
h63	1		V	I	D	L	P	F	.	K	S	N	K	A	R	S	R	Y	.	K	G	Y	K	H	K	Y	K	H	K	F	26
h64	1		V	I	D	L	P	F	.	K	S	.	.	.	.	.	.	.	.	K	G	Y	K	H	K	Y	K	H	K	F	19
h65	1		V	I	D	L	P	F	.	.	.	N	K	A	R	S	R	Y	H	K	G	Y	K	H	.	Y	K	H	K	F	24
h66	1		V	I	D	L	P	F	.	.	.	N	.	A	R	S	R	Y	H	K	G	Y	K	H	K	Y	K	H	K	F	24
h67	1		V	I	D	L	P	F	.	.	S	N	K	A	R	S	R	Y	H	K	G	Y	K	H	K	Y	K	H	K	F	26
h68	1		V	I	D	L	P	F	.	X	X	N	K	A	R	S	R	Y	H	K	G	Y	K	H	.	.	.	H	K	F	22
h69	1		V	I	D	L	P	F	.	X	X	N	K	A	R	S	R	Y	H	K	G	Y	K	H	.	Y	.	H	K	F	23
h70	1		V	I	D	L	P	F	N	K	S	N	K	A	R	S	.	.	.	K	G	Y	K	H	K	Y	K	H	K	F	25
h71	1		V	I	D	L	P	F	N	K	S	N	K	A	R	S	R	Y	H	K	G	.	.	R	K	Y	K	H	K	F	26
h72	1		V	I	D	L	P	F	N	K	S	N	K	A	R	S	R	Y	H	K	G	Y	K	H	K	Y	K	H	K	.	27
h73	1		V	I	D	L	P	F	N	K	S	N	K	A	R	S	R	Y	H	K	G	Y	K	H	K	Y	K	.	K	.	26
h74	1		V	I	D	L	P	F	N	K	S	N	K	A	R	S	R	Y	H	K	G	Y	K	H	K	Y	K	.	.	F	26
h75	1		V	I	D	L	P	F	N	K	S	N	K	A	R	S	R	Y	H	K	G	Y	K	H	.	.	K	H	K	.	25
h76	1		V	I	D	L	P	F	N	K	S	N	K	A	R	S	R	Y	H	K	G	Y	K	H	.	Y	K	H	K	F	27
h77	1		V	I	D	L	P	F	N	K	S	N	K	A	R	S	R	Y	H	K	G	Y	K	H	X	X	X	X	K	F	24
h78	1		V	I	D	L	P	F	N	K	S	N	K	A	R	S	R	Y	H	K	G	Y	.	H	.	.	.	.	.	.	21
h79	1		V	I	D	L	P	F	N	K	S	N	K	A	R	S	R	Y	H	K	G	Y	.	.	.	Y	K	H	K	F	25
h80	1		V	I	D	L	P	F	N	K	S	N	K	.	.	.	.	.	.	K	G	Y	K	H	K	Y	K	H	K	F	22
h81	1		V	I	D	L	P	F	N	K	S	N	K	.	R	S	R	Y	H	K	G	Y	K	H	K	Y	K	H	K	F	27
h82	1		V	I	D	L	P	F	N	K	S	N	.	A	R	S	R	Y	H	K	G	Y	K	H	K	Y	K	H	K	F	27
h83	1		V	I	D	L	P	F	N	K	S	N	.	.	R	.	.	.	.	K	G	Y	K	H	K	Y	K	H	K	F	22
h84	1		V	I	D	L	P	F	N	K	S	.	K	.	.	.	.	Y	.	K	G	Y	K	H	K	Y	K	H	K	F	22

© 2022 Zhao LP et al. *JAMA Network Open.*

h85	1		V	I	D	L	P	F	N	.	.	.	.	.	.	.	.	.	.	K	G	Y	K	H	K	Y	K	H	K	F	18
h86	1		V	I	D	L	P	F	N	.	.	.	.	K	.	.	.	Y	.	K	G	Y	K	H	K	Y	K	H	K	F	20
h87	1		V	I	D	L	P	F	N	.	.	.	.	K	R	S	.	Y	H	K	G	Y	K	H	K	Y	K	H	K	F	23
h88	1		V	I	D	L	P	F	N	.	S	N	K	A	.	.	.	.	.	K	G	Y	K	H	K	Y	K	H	K	F	22
h89	1		V	I	D	L	P	F	N	.	S	N	K	A	R	S	R	Y	.	K	G	Y	K	H	K	Y	K	H	K	F	26
h90	1		V	I	D	L	P	.	.	.	.	N	K	A	R	S	R	Y	H	K	G	Y	K	H	K	Y	K	H	K	F	24
h91	1		V	I	D	L	P	.	N	K	S	N	K	A	R	S	R	Y	H	K	G	Y	K	H	K	Y	K	H	K	F	27
h92	1		V	I	D	L	P	.	N	.	S	N	K	A	.	S	.	.	H	K	G	Y	.	R	.	.	.	.	.	.	16
h93	1		V	I	D	L	.	.	.	K	S	N	K	A	R	S	R	Y	H	K	G	Y	K	H	K	Y	K	H	K	F	25
h94	1		V	I	D	L	.	.	N	.	S	.	K	A	.	.	.	.	H	K	G	Y	K	R	.	.	.	.	.	.	14
h95	1		V	I	D	L	.	.	X	X	X	.	.	.	R	S	R	Y	H	K	G	Y	.	.	X	Y	K	H	K	F	17
h96	1		V	I	D	P	P	F	N	.	S	N	K	A	.	.	.	.	.	K	G	Y	K	H	K	Y	K	H	K	F	22
h97	1		V	I	D	P	.	F	N	K	S	.	.	.	.	.	.	.	.	K	G	Y	K	H	K	Y	K	H	K	F	19
h98	1		V	I	D	P	.	.	N	.	.	.	K	.	.	.	.	.	.	K	G	.	K	H	K	.	.	.	.	.	11
h99	1		V	I	D	.	P	F	N	K	S	N	K	A	R	S	R	Y	H	K	G	Y	.	R	.	.	.	.	.	.	20
h100	1		V	I	D	.	.	.	.	K	.	N	K	A	R	S	R	.	H	K	G	Y	K	H	K	Y	K	H	K	F	22
h101	1		V	I	D	.	.	.	.	K	S	N	K	A	R	.	.	Y	.	K	G	Y	K	H	K	Y	K	H	K	F	21
h102	1		V	I	D	.	.	.	.	K	S	.	K	A	R	S	R	Y	H	K	G	Y	K	H	K	Y	K	H	K	F	23
h103	1		V	I	D	.	.	.	.	.	.	.	K	A	.	.	.	.	.	K	G	Y	K	H	K	Y	K	H	K	F	16
h104	1		V	I	D	.	.	.	.	.	S	N	K	A	R	S	R	Y	H	K	G	Y	K	H	K	Y	K	H	K	F	23
h105	1		V	I	D	.	.	.	N	.	.	N	K	.	.	.	.	.	H	K	G	.	.	R	.	.	.	.	.	.	10
h106	1		V	I	D	.	.	.	N	.	.	N	K	.	.	S	.	.	H	K	G	.	.	R	.	.	.	.	.	.	11
h107	1		V	I	D	.	.	.	N	.	.	N	K	.	.	S	.	.	.	K	G	.	.	R	.	.	.	.	.	.	10
h108	1		V	I	D	.	.	.	N	.	.	.	K	A	.	.	.	.	.	K	G	Y	.	R	.	.	.	.	.	.	10
h109	1		V	I	D	.	.	.	N	.	.	.	K	.	.	.	.	.	.	K	G	Y	K	H	K	Y	K	H	K	F	16
h110	1		V	I	D	X	X	X	.	K	S	N	K	A	R	S	R	Y	H	I	G	Y	K	H	.	.	K	H	X	X	20
h111	1		V	I	.	L	P	F	N	K	S	N	K	A	R	S	R	Y	H	K	G	Y	.	.	K	Y	K	H	.	F	24
h112	1		V	I	.	L	P	F	N	K	S	N	K	A	R	S	R	Y	.	K	G	Y	K	H	K	Y	K	H	K	F	26
h113	1		V	I	.	.	P	.	N	.	S	N	K	A	R	S	.	Y	H	K	G	.	K	H	K	Y	.	.	.	.	18
h114	1		V	I	.	.	.	.	.	K	.	.	K	.	.	.	.	.	.	K	G	Y	K	H	K	Y	K	H	K	F	15
h115	1		V	I	.	.	.	.	.	K	S	N	K	A	.	.	.	.	.	K	G	Y	K	H	K	Y	K	H	K	F	18

© 2022 Zhao LP et al. *JAMA Network Open.*

h116	1		V	I	.	.	.	.	.	K	S	N	K	A	R	S	R	.	.	K	G	Y	K	H	K	Y	K	H	K	F	21
h117	1		V	I	.	.	.	.	.	K	S	.	K	.	.	.	.	.	.	K	G	Y	K	H	K	Y	K	H	K	F	16
h118	1		V	I	.	.	.	.	.	.	.	.	K	A	R	.	R	.	.	K	G	Y	K	H	K	Y	K	H	K	F	17
h119	1		V	I	.	.	.	.	.	.	.	.	K	.	.	S	.	.	.	K	G	.	K	H	K	Y	K	.	K	.	12
h120	1		V	I	.	.	.	.	.	.	.	.	.	.	.	.	.	.	.	K	G	Y	K	H	K	Y	K	H	K	F	13
h121	1		V	I	.	.	.	.	.	.	.	.	.	K	.	.	.	Y	.	K	G	Y	K	H	K	Y	K	H	K	F	15
h122	1		V	I	.	.	.	.	.	.	S	N	K	A	.	.	.	.	.	K	G	Y	K	H	K	Y	K	H	K	F	17
h123	1		V	I	.	.	.	.	.	.	S	.	.	.	R	.	R	Y	H	K	G	Y	K	H	K	Y	K	H	K	F	18
h124	1		V	I	.	.	.	.	N	K	S	.	K	.	.	.	.	.	.	.	G	Y	.	R	.	Y	K	.	.	.	11
h125	1		V	I	.	.	.	.	N	.	S	N	K	A	R	S	R	Y	H	K	G	Y	K	H	K	Y	K	H	K	F	23
h126	1		V	I	.	.	.	.	N	X	.	.	.	K	.	.	.	Y	.	X	G	Y	K	H	X	.	X	H	K	F	12
h127	1		V	I	.	X	X	X	X	.	.	.	K	.	.	.	.	.	.	K	G	Y	.	H	K	Y	.	H	K	F	12
h128	1		V	I	.	X	X	X	X	X	X	X	X	X	X	X	X	X	X	K	G	Y	.	.	K	Y	K	H	K	F	11
h129	1		V	I	X	L	.	.	.	.	.	.	K	.	R	S	R	Y	H	K	G	X	.	.	X	Y	K	H	K	F	16
h130	1		V	I	X	X	.	.	.	.	.	.	K	.	R	S	R	Y	H	X	G	Y	.	.	.	.	.	H	.	.	11
h131	1		V	I	X	X	X	X	X	X	X	X	X	X	X	X	X	X	X	K	G	Y	K	H	K	Y	.	H	K	F	12
h132	1		V	I	X	X	X	X	X	X	X	X	X	X	X	X	X	X	X	K	G	Y	K	H	X	Y	K	H	K	F	12
h133	1		V	I	X	X	X	X	X	X	X	X	X	X	X	X	X	X	X	K	G	Y	.	.	.	Y	K	H	K	F	10
h134	1		V	.	D	F	P	F	N	K	.	N	K	A	R	.	R	Y	H	.	G	.	.	R	.	.	.	.	.	.	16
h135	1		V	.	D	L	.	.	N	.	.	N	K	.	.	S	.	.	.	K	G	Y	.	R	.	.	.	.	.	.	11
h136	1		V	.	.	P	.	F	N	.	S	.	K	.	.	S	.	.	H	K	G	.	.	R	.	Y	.	.	.	.	12
h137	1		X	X	D	L	P	F	N	K	S	N	K	A	R	S	.	Y	H	K	G	Y	K	R	.	.	.	.	.	.	19
h138	1		X	X	D	L	P	F	N	K	S	N	K	A	R	S	R	Y	H	K	G	Y	K	H	X	X	X	X	X	X	20
h139	1		X	X	D	L	P	F	N	K	S	N	K	A	R	S	R	Y	H	K	G	Y	K	H	X	Y	K	H	K	F	25
h140	1		X	X	D	L	P	F	N	K	S	N	K	A	R	S	R	Y	H	K	G	Y	.	R	.	.	.	.	.	.	19
h141	1		X	X	D	L	P	.	N	.	S	N	K	A	R	S	R	Y	H	K	G	Y	K	H	.	.	.	.	.	.	18
h142	1		X	X	.	.	.	.	.	.	.	.	K	.	.	.	.	.	.	.	G	.	K	H	K	Y	K	H	K	F	10

© 2022 Zhao LP et al. *JAMA Network Open.*

eFigure 1. Heatmap Representation of Polymutant Expansions in Zambia

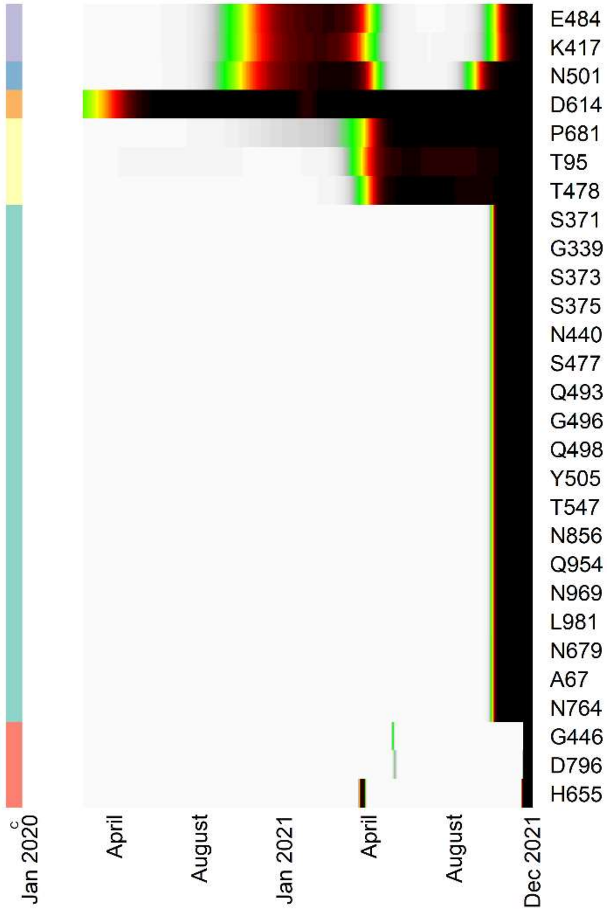

© 2022 Zhao LP et al. *JAMA Network Open.*

eFigure 2. Illustration on Negative Estimate of Detection Time Due to Sparse Collection of Viral Sequences During a Critical Period When a Polymutant Rises Rapidly to Become a Dominant Mutation in a Short Period of Time

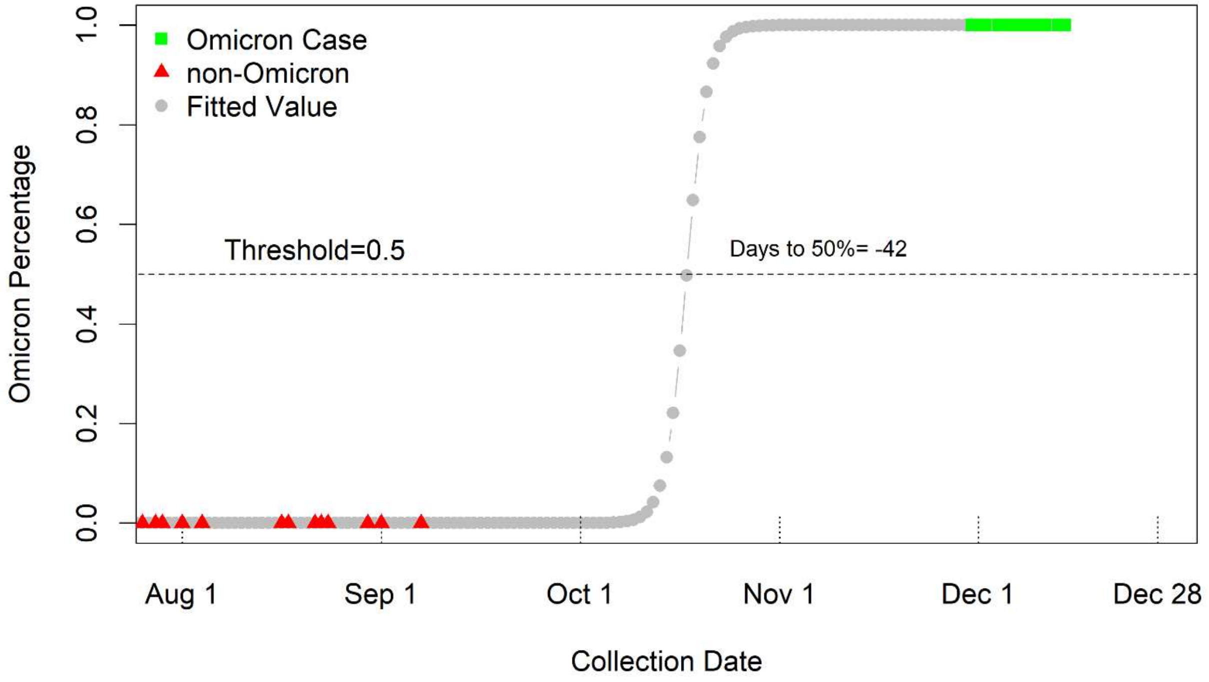

© 2022 Zhao LP et al. *JAMA Network Open.*

eReferences.

1. Katoh, K., and Standley, D.M. (2013). MAFFT multiple sequence alignment software version 7: improvements in performance and usability. Mol Biol Evol *30*, 772-780. 10.1093/molbev/mst010.
2. Nakamura, T., Yamada, K.D., Tomii, K., and Katoh, K. (2018). Parallelization of MAFFT for large-scale multiple sequence alignments. Bioinformatics *34*, 2490-2492. 10.1093/bioinformatics/bty121.
3. Zheng, X. (2018). Imputation-Based HLA Typing with SNPs in GWAS Studies. Methods Mol Biol *1802*, 163-176. 10.1007/978-1-4939-8546-3_11.
4. Li, S.S., Wang, H., Smith, A., Zhang, B., Zhang, X.C., Schoch, G., Geraghty, D., Hansen, J.A., and Zhao, L.P. (2011). Predicting multiallelic genes using unphased and flanking single nucleotide polymorphisms. Genetic epidemiology *35*, 85-92. 10.1002/gepi.20549.
5. Hastie, T., and Tibshirani, R. (1991). Generalized Additive Models. Statistical Science *1*, 297-318.
6. Efron, B., and Tibshirani, R. (1998). Cross-validation and the bootstrap: estimating the error rate of a prediction rule.
7. Murtagh, F., and Legendre, P. (2014). Ward's Hierarchical Agglomerative Clustering Method: Which Algorithms Implement Ward's Criterion? J Classif *31*, 274-295. 10.1007/s00357-014-9161-z.

© 2022 Zhao LP et al. *JAMA Network Open.*
